# Endogenous Small-Noncoding RNAs and Potential Functions in Desiccation Tolerance in *Physcomitrella Patens*

**DOI:** 10.1038/srep30118

**Published:** 2016-07-22

**Authors:** Jing Xia, Xiaoqin Wang, Pierre-François Perroud, Yikun He, Ralph Quatrano, Weixiong Zhang

**Affiliations:** 1 Institute for Systems Biology, Jianghan University, Wuhan, Hubei 430056, China; 2 Department of Computer Science and Engineering, Washington University, St. Louis, MO 63130, USA; 3 College of Life Sciences, Capital Normal University, Beijing 100048, China; 4 Department of Biology, Washington University, St. Louis, MO 63130, USA; 5 Department of Genetics, Washington University, St. Louis, MO 63130, USA

## Abstract

Early land plants like moss *Physcomitrella patens* have developed remarkable drought tolerance. Phytohormone abscisic acid (ABA) protects seeds during water stress by activating genes through transcription factors such as ABSCISIC ACID INSENSITIVE (ABI3). Small noncoding RNA (sncRNA), including microRNAs (miRNAs) and endogenous small-interfering RNAs (endo-siRNAs), are key gene regulators in eukaryotes, playing critical roles in stress tolerance in plants. Combining next-generation sequencing and computational analysis, we profiled and characterized sncRNA species from two ABI3 deletion mutants and the wild type *P. patens* that were subject to ABA treatment in dehydration and rehydration stages. Small RNA profiling using deep sequencing helped identify 22 novel miRNAs and 6 genomic loci producing trans-acting siRNAs (ta-siRNAs) including TAS3a to TAS3e and TAS6. Data from degradome profiling showed that ABI3 genes (*ABI3a*/*b*/*c*) are potentially regulated by the plant-specific miR536 and that other ABA-relevant genes are regulated by miRNAs and ta-siRNAs. We also observed broad variations of miRNAs and ta-siRNAs expression across different stages, suggesting that they could potentially influence desiccation tolerance. This study provided evidence on the potential roles of sncRNA in mediating desiccation-responsive pathways in early land plants.

Early land plants such as moss *Physcomitrella patens* have developed remarkable drought tolerance for survival, whereas modern vascular plants have only retained drought tolerance in a few specialized tissues like seeds and spores^1^. During seed development, an essential plant phytohormone abscisic acid (ABA) protects seeds by inducing a variety of genes including those encoding proteins for seed storage as well as genes that encode late embryogenesis abundant (LEA) proteins^2^,^3^. ABA is prevalent in the developmental stages of land plants and plays a critical role in mediating plant growth and development as well as promoting desiccation tolerance^4^. Abundant ABA typically leads to arrest of growth under abiotic stress conditions^5^.

ABA activates genes that have stress-tolerance functions through transcription factors such as ABSCISIC ACID INSENSITIVE 3 (*ABI3*)^6^. In *P. patens*, deletion mutants of *ABI3* are not able to recover from desiccation despite being treated with ABA, indicating that *ABI3* is required for *P. patens* vegetative tissues to survive desiccation^1^. The role of *ABI3* has been postulated to maintain the expression of ABA-inducible genes in recovery stages during rehydration, as the loss of *PpABI3* has little effect on most ABA up-regulated genes before rehydration. For instance, ABA induces *lea group3* which remains expressed after a 24-hour ABA treatment and a 24-hour dehydration in both wild-type (WT) and ABI3 deletion mutants. In the WT, *lea group3* maintains its expression after 5 and 15-minute rehydrations but exhibits dramatically reduced expression levels in the *ABI3* mutants during rehydration^1^, indicating its dependence on the activity of *ABI3*. Transcription factors *ABI3* and *ABI5* are known regulators of ABA responses in *Arabidopsis thaliana* germination^7^,^8^.

*P. patens* is capable of producing small RNAs, including microRNAs (miRNAs) and endogenous small-interfering RNAs (endo-siRNAs)^9^. Plant miRNAs are normally derived from hairpin-shaped structured pre-miRNAs which are processed by Dicer-like (DCL) proteins in the nucleus to release ~22-nt double-stranded RNAs with ~2-nt 3′ overhangs, namely miRNA/miRNA* duplexes. Mature miRNAs are then loaded into the RNA-induced silencing complexes (RISC) that contain Argonautes (AGO) in the cytoplasm in order to exert their regulatory effect by guiding the RISC to target transcripts through complete or partial complementary base pairing^10^. Endogenous small interfering RNAs (endo-siRNAs) can arise from long double stranded RNAs (dsRNAs) of overlapping antisense transcripts or from products of RNA-dependent RNA polymerases (RdRP)^11^,^12^,^13^,^14^. In plants, different classes of endo-siRNAs have been described based on their distinct characteristics, biogenesis pathways, and functions^15^,^16^. For example, noncoding TAS genes generate trans-acting siRNAs (ta-siRNAs)^17^,^18^,^19^,^20^ by consecutive Dicer processing in a phased fashion^10^. In addition, long hairpins are prevalent and have the capacity to host both miRNAs and siRNAs^21^. While DCL1 processes multiple phased miRNAs on long hairpin substrates, the other Dicer-like enzymes (DCL2, DCL3, and DCL4) are able to process long hairpins which give rise to endo-siRNAs of various sizes^22^.

It has been shown that miRNAs play important roles in gene regulation in response to ABA treatment in plants. In germinating *A. thaliana* seeds, ABA induces accumulation of miR159 in an *ABI3*-dependent fashion and miR159 mediates the cleavage of two transcription factors MYB101 and MYB33 *in vivo*^5^. Additional evidence has indicated that miR159 functions through a homeostatic mechanism to maintain sensitivity to hormone signaling during seedling stress responses^5^. However, miR159 is not encoded in moss. Instead, miR319 is known to regulate MYB genes in moss^9^. Interestingly, miR477h has been predicted to target ABI3 gene^9^. In *P. patens*, miRNAs have been shown to induce hypermethylation of their target genes and consequential transcriptional silencing upon ABA treatment^23^. For example, ABA-induced accumulation of miR1026 subsequently leads to hypermethylation and down-regulation of the *PpbHLH* gene, indicating that miRNAs also function in epigenetic control of stress-responsive genes^23^. Like miRNAs, endo-siRNAs in plants also play important roles in gene regulation. Trans-acting siRNAs derived from well-conserved TAS3 genes regulate auxin response factors (ARF) in plants^17^.

Despite the known gene regulatory activities of miRNAs triggered by ABA, most of their functions have yet to be analyzed. We performed a comprehensive study of small-noncoding RNAs in response to ABA in *P. patens* through Next-Generation Sequencing (NGS) based profiling of small RNA species. We compared the WT and the ABI3-deleted mutants in ABA treated and untreated tissues as well as in dehydration and rehydration stages. The results showed that moss ABI3 genes (*ABI3a*/*b*/*c*) were potentially regulated by miR536, a basal plant-specific miRNA that is conserved but not beyond *Selaginella moellendorffii*^9^ in the green plant lineage. We also identified novel miRNAs and endo-siRNAs derived from long hairpin RNAs, miRNA-cleaved transcripts, and putative target genes of small RNAs by investigating data from degradome profiling of *P. patens*. Differential expression analyses revealed a broad alteration of small RNA expressions across different conditions, indicating the involvement of small RNAs in the plant’s adaptation to dynamic environmental changes.

## Results

### Profiling of small-noncoding RNAs in ABA-treated and dehydrate mosses

Eight small-RNA libraries from normal and ABI3 deletion mutant *P. patens* at 7-day ABA-treated, dehydrated, and then rehydrated stages were prepared and sequenced through NGS using the Illumina Genome Analyzer IIx (GAIIx) (Table S1). These libraries produced more than 190 million raw sequencing reads total, among which 108 million (~57% of the total) are high-quality, adapter-trimmed reads (qualified reads). A total of 65% of the qualified reads can map to the *P. patens* reference genome (http://www.phytozome.net) allowing no mismatches (Table S1).

### miRNAs regulate ABA responsive genes

**Figure 1:**
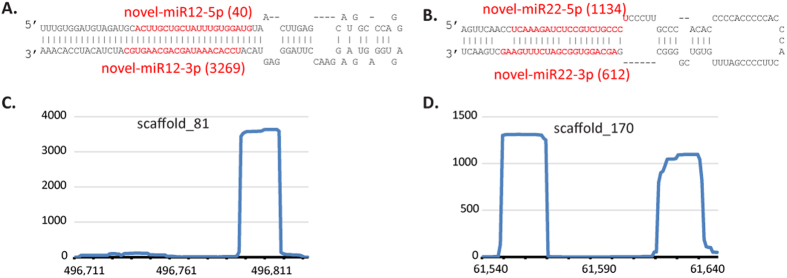
Examples of two novel miRNAs in *P. patens*. The hairpin structures of novel-miR12 (**A**) and novel-miR22 (**B**) are shown with mature miRNA-5p and miRNA-3p highlighted in red. The mature miRNAs form RNA duplexes with ~2-nt 3′-overhangs. The number in parenthesis represents the number of reads from sequencing profiling. The genomic loci for novel-miR12 (**C**) and novel-miR22 (**D**) and the read distributions across the two loci.

NGS profiling detected a total of 226 miRNAs in the conditions we examined (see Methods). Among the expressed miRNAs, we found 22 novel miRNAs (Table S2) and 204 of the 229 *P. patens* miRNAs that are currently annotated in miRBase (version 21, Tables S2 and S3). Two novel miRNAs are shown in Fig. 1 with their characteristic hairpin structures and RNA-RNA duplexes with ~2-nt 3′ overhangs. We failed to find homologous hairpin sequences of the 22 novel miRNAs in other plant species using conservation analysis, which indicates that these novel miRNAs may be *P. patens* specific.

The overall abundance of the detected miRNAs amounted to around 7 million reads, accounting for ~5.6% of the total genome-mapped, qualified reads. This ratio of miRNA abundance is similar to that in rice (5.2%), but less than in Arabidopsis (14%)^24^. Overall, the 5 most abundant mature miRNAs were miR156, miR319, miR535, miR904, and miR1028 (Table S3). The most abundant novel miRNA, novel-miR12, has more than 3 K reads in all four libraries combined (Table S2), making it moderately abundant among all 226 detected miRNAs.

**Figure 2:**
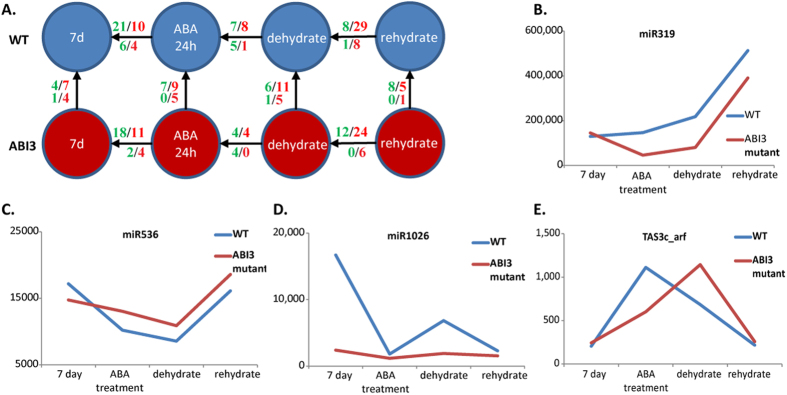
(**A**) The number of differentially expressed miRNAs in various comparisons. The arrow indicates the comparison direction (source as case and destination as control). The numbers on an edges are the number of upregulated sncRNAs (green) and the number of downregulated sncRNAs (red). The first row is the number of the DE miRNAs and the second row is the number of the DE siRNAs. A large amount of DE miRNAs were observed after ABA treatment for 24 h versus the control, and rehydration versus dehydration stages. (**B** to **D**) Normalized digital expression levels of miRNAs at four different conditions for the WT and mutant.

The expression of detected miRNAs indicated their roles in desiccation tolerance. A substantial number of miRNAs exhibited dramatic variations in expression levels across different conditions. Overall, 46 expressed miRNAs were differentially expressed (DE) by at least 2-fold in two different conditions (Fig. 2A and Table S4). On the other hand, only a few miRNAs were differentially expressed during dehydration versus ABA-treatment in both the WT and mutant, indicating that miRNAs are less perturbed under dehydration stress after ABA treatment. These observations are consistent with changes in protein-coding gene expressions, as ABA-induced genes have been shown to maintain their expression in a relatively stable fashion during the dehydration stage^1^. In contrast, a large number of miRNAs were DE during rehydration in both the WT and mutants (Fig. 2A), suggesting that miRNAs are involved in the rehydration process.

miRNAs directly regulate several genes that are critical for ABA responses, including ABA receptors^25^ and those that are induced by ABA^1^. We identified a total of 230 miRNAs binding sites on 35 ABA-relevant genes using computational methods (see Methods, Table S5). The most significant of these putative target genes were those related to ABA responses, particularly the genes in the ABI3, MYB and LEA families that are known to protect the seed during desiccation. A total of 22, 5, and 20 miRNAs targeted ABI3, MYB, and LEA genes, respectively (Table S5).

**Figure 3:**
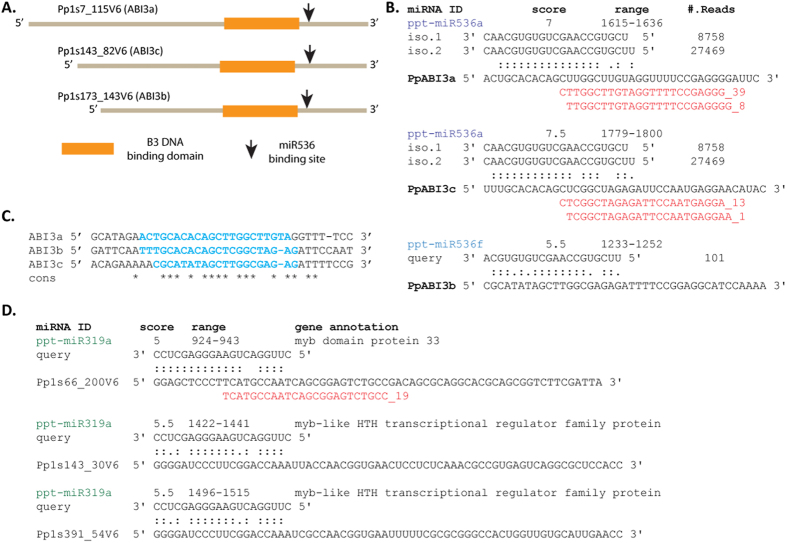
The binding sites of miRNA on moss genes. (**A**) Three ABI3 genes are annotated with coding regions (CDS) consisting of B3 DNA binding domain, while miR536 targeting sites are downstream to the domain at each genic locus. (**B**) Base pairings between miR536 (query) and ABI3 (target) are shown in the format of “:” as a canonical match, “ . ” as a wobble match, and blank space as a mismatch. The column of “range” indicates the start and end position of a binding site on a transcript. miR536 sequences with alternative 5′-ends were plotted from 3′ to 5′, and #. Reads are listed for each miR536 isoform. Degradome reads corresponding to miRNA cleavages were highlighted in red. (**C**) Sequence conservation among the binding sites of ABI3 genes, where * represents a conserved site of the three genes. (**D**) Base pairings between members of the miR319 family (query) and three MYB genes (target).

miR536 may potentially cleave the genes in the ABI3 family. ABI3 has three paralogs in *P. patens* (i.e. ABI3a/b/c) and the highly expressed miR536 family bound to all three paralogs at specific loci which exist downstream of the B3 domain of the ABI genes (Fig. 3A). As shown, a sufficient number of reads from RNA degradation product profiling (i.e. degradome data GSE16367) were uniquely mapped to the binding regions of ABI3a and ABI3c. Note that the 5′-ends of the degradome reads were enriched at the canonical miRNA cleavage sites, indicating that miR536 endogenously cleaves the ABI3 genes upon binding (Fig. 3B). The three binding sites showed strong sequence conservation at the cleavage sites but displayed weak conservation outside of the miRNA binding regions (Fig. 3C). miR536 is a basal plant-specific miRNA previously identified in moss (*P. patens*) and lycopod (*S. moellendorffii*). It was highly abundant in our current profiling data, thus suggesting that the members of the miR536 family behave as genuine regulators of ABA responses.

miR319 is known to target three paralogous MYB genes in moss, including the two paralogs, Pp1s143_30V6.1 and Pp1s391_54V6.1^9^, and one target we newly identified in our study, Pp1s66_200V6.1. Some of the reads from the degradome profiling were aligned to the miR319 cleavage site on the new miR319 target (MYB, Pp1s66_200V6.1, Fig. 3D). Importantly, miR319 was highly expressed in the current sequencing data and exhibited down-regulation (3.2-fold) in the ABI3 knockout mutant. However, it also displayed a 1.3-fold up-regulation in the WT, suggesting that miR319 responded to ABA-treatment in the mutants without the ABI3 genes expressed. Taken together, we identified miRNAs as regulators of ABA-related genes in moss, which indicates that the earliest land plants developed miRNA regulatory mechanisms to tolerate desiccation.

Additional analysis identified putative targets of novel miRNAs with signature reads of small RNA cleavage activities captured in the degradome data. In total, 6 miRNA binding sites on 6 transcripts exhibited cleavage signals (File S1). For example, sufficient degradome reads can be perfectly mapped to the predicted binding site on Pp1s173_12V6.1, a SER/ARG-rich gene. The 5′-ends of some aggregated reads aligned on the 11-th nucleotide of the novel-miR21 (counting from the right of the query sequence in Figure S1). Furthermore, the reads were only enriched at the miRNA cleavage site, which suggests that novel-miR21 can bind to and cleave the target transcript.

### Endogenous siRNAs and their role in desiccation tolerance

A genome-wide search for putative siRNA-yielding loci found 6 loci producing trans-acting siRNAs (ta-siRNAs) in moss including TAS3a to TAS3e as well as TAS6 (Table S6)^17^. These results show the robustness and high sensitivity of the method in finding ta-siRNAs (see Methods). We also identified two long hairpin structures which can potentially produce siRNAs. The two hairpins are extensively long, with around 180 and 370 base pairs, and contain highly-annealed stems (Figures S2 and S3). The majority of reads were uniquely aligned, which indicates that small RNAs were processed and derived exclusively from the loci. For example, a total of 6,761 reads were uniquely aligned to hpRNA-1 (93% are 20- to 22-nt), with significantly more reads arising from the 3′-arm than the 5′-arm of hpRNA-1 (with around 5-fold difference). Interestingly, more reads were mapped to the basal than the loop region of the stem (Figure S2), which indicates a base to loop endonuclease processing along the hairpin substrate^26^. Long hairpins have been observed to undergo transitional stages in evolution and are imprecisely processed where small RNAs are produced by one or more of the siRNA-generating DCL enzymes^21^. This suggests that the small RNAs that are derived from the two long hairpins are likely to be endo-siRNAs.

Some siRNAs were also differentially expressed across conditions (Fig. 2A). For example, the ta-siARF species from TAS3 genes was up-regulated upon ABA-treatment in both the WT and mutants (Fig. 2E), potentially having an impact on the downstream targets including auxin response factor (ARF). This is in agreement with another ARF-regulator miRNA, miR393 (not encoded in moss), which was upregulated in Arabidopsis^27^ and rice^28^ under drought stress.

Furthermore, degradome profiling can also help detect siRNA cleavage events, exemplified by several known ta-siRNAs in Arabidopsis^29^,^30^. Here, we utilized the degradome data to identify targets of highly expressed siRNAs derived from long hairpins and TAS transcripts (see Methods). We detected 22 targets for the set of siRNAs, listed in File S2, along with functional annotations and alignment of reads from degradome profiling. One example is the siRNA species from hpRNA-2, which putatively targets and cleaves a gene encoding ribosomal protein S17, i.e., Pp1s76_75V6.1 (Figure S4). This result shows that these siRNAs have the potential of regulating cellular genes.

## Discussion

ABA is a potent regulator of abiotic stress signaling in plants including moss, and ABA pretreatment is required for moss (*P. patens*) to confer desiccation tolerance^31^. In our study, we showed that this desiccation tolerance in moss was in large part attributed to small RNA, particularly miRNA mediated gene expression regulation.

Our results showed that the miR536 family functioned as regulators of the ABI3 genes on the ABA signaling pathway. We hypothesize that the acquisition of miR536 regulation on ABA signaling contributes significantly to the vegetative desiccation tolerance in moss, a trait that vascular plants do not possess. Several lines of evidence support this hypothesis. First, *P. patens* carries three ABI3 paralogs, while other vascular plants contain a single copy of this critical gene^32^. Second, there are 6 paralogous copies of miR536 in *P. patens*, while there is only one copy of miR536 in *Selaginella moellendorffii* and miR536 is completely missing beyond bryophytes. In other words, compared to other plant species *P. patens* contains a larger number of ABI3 and miR536 paralogous genes. This discrepancy may be attributed to the robustness and complexity of ABA signaling in moss and contribute to vegetative desiccation tolerance in the plant. Third, a conservation analysis of miRNA regulation of ABI3 genes in lycopod (*Selaginella moellendorffii*), rice (*Oryza sativa*), and *Arabidopsis thaliana* showed that the regulation of ABI3 genes by miRNAs is missing in these species (data not shown). As a previous study has shown, miR536 in *P. patens* directly cleaves the F-box genes^9^, which are auxin receptors^33^. Due to the abundant number of ABI3 genes and miR536 members in *P. patens* as well as desiccation tolerance of all moss vegetative and haploid tissues, we conclude that each ABI3 gene and each member of the miR536 family are involved in different ABA signaling pathways in each tissue. These results also indicate that these genes might have contributed to plant early adaptation to harsh land environments, which was later recognized to link to the seed strategy for survival on land for Angiosperms. Since ABI3 genes are a major regulator of gene expression in the seeds of most vascular plants which lack miR536, our results also suggest that higher plants must have introduced alternative miRNA strategies on ABI3 regulation, for example through miR159 regulation on ABI3 in Arabidopsis^5^.

Finally, recent studies also showed that ABA treatment induced overexpression of miR1026 and consequentially led to the down-regulation of PpbHLH gene, a target of miR1026^23^. In addition, PpbHLH has been experimentally validated as a target of miR902-5p^9^, indicating that PpbHLH is tightly regulated by multiple miRNAs. miR1026 was highly expressed in our sequencing data and was dramatically down-regulated by more than 8-fold in the WT after 24-hour ABA-treatment (Table S4) but not in the mutant, which indicates a large alteration to the gene regulatory circuit involving ABA, PpbHLH and miR1026.

Targets of miRNAs in moss have been studied using both bioinformatic and experimental methods^34^,^35^,^36^,^37^. Most bioinformatic approaches follow restrictive rules focusing mainly on misalignments between a miRNA and a target. As a result, these methods are not as effective as expected. Indeed, recent studies have revealed noncanonical target sites of plant miRNAs which are oversighted in the original studies^38^,^39^. These non-canonical binding sites form unconventional complementary sequences to miRNA sequences but with evident miRNA cleavage activities. For example, miR398 cleaves blue copper-binding protein (BCBP) at a non-canonical complementary site in the 5′-untranslated region (UTR) of BCBP with a bulge of six nucleotides opposite to the 5′ region of the miRNA^39^. In our current study, the target sites of miR536 on ABI3 genes showed atypical annealing (Fig. 3), as there are two mismatches and one wobble match occurring from the 2-nd to 8-th nucleotide (the so called seed region of a miRNA in animal). We also observed a low degree of complementarity within the seed region in the binding of miR319 and MYB gene (Fig. 3D), whereas experiments demonstrate that miR319 is able to cleave MYB gene in moss^9^. Taken together, our results suggest a broad existence of non-canonical target sites of plant miRNAs that may not have a high degree of complementarity within seed regions.

## Materials and Methods

### Plant materials

*Physcomitrella patens* subspecies patens (Gransden) was used as the wild type (WT) strain. The ABI3 deletion mutants were two independent lines (*∆abi3-1* and *∆abi3-2*) in which all three *P. patens* ABI3 genes (A, B and C) were deleted (see ref. 1 for details). The WT and mutants were cultured at 25 °C under a 16 h light and 8 h dark regime. Under these conditions, the protonemal filaments are not under water stress. For ABA treatments, protonemal tissue grown on cellophane was transferred to BCDA medium containing 50 μM ABA and incubated for the indicated times.

### Desiccation assay

Protonemal tissues grown for 7 days under standard conditions (on cellophane in direct contact with the medium) are reflective of growth under non-stressed conditions. Protonemal tissues grown on a cellophane were subjected to water stress by transferring onto a filter paper (Whatman, USA) in an empty Petri dish and desiccating inside a laminar flow hood for 24 h. The desiccated tissues were transferred to BCDA plate and 2 mL of sterile water or different concentrations of ABA were added, similar to the protocol used for the moss Funaria. These rehydrated tissues were then incubated under standard growth conditions described above.

### RNA profiling using NextGen deep sequencing

RNA from protonemal tissus of the WT and mutant lines were grown for 7 days then subjected to 24 hours of ABA treatment, dehydration, and rehydration (see Fig. 2A). These samples were extracted separately, following the protocol in^1^. The protonemal tissues from the two mutant lines at corresponding stages were mixed before RNA extraction. Total RNA was isolated using the Plant RNA Isolation Mini kit (Agilent Technologies, Inc., DE, USA) following the manufacturer’s instructions. Total RNA (1 μg) was reverse transcribed by ThermoScript™ RT-PCR System (Invitrogen Co., CA, USA) with oligo-dT(20) primer following the manufacturer’s instruction. PCR was carried out using 1 μL of cDNA with GoTaq^®^ Green Master mix (Promega Co., WI, USA). Small-RNA libraries were prepared for Illumina sequencing following the manufacturer’s instructions^40^. miRNeasy Mini Kit (Qiagen) was used for RNA extraction, with on-column DNase I digestion, followed by sequencing on the Illumina GAIIx platform using Small RNA Sample Prep Kit (Illumina).

### Preprocessing of sequencing data

Raw sequence reads that contained no 3′ sequencing adaptor, were of low quality, or were shorter than 17-nt were discarded. The adaptor trimming was done by an in-house method that recursively searches for the longest substring of the adaptor appearing within a sequence read. If a raw sequence read did not have a substring of the adaptor longer than 6-nt, it was considered to carry no adaptor. The adaptor-trimmed sequences with no ambiguous reads, which were referred to as qualified reads, were then mapped to the *P. patens* reference genome using Bowtie^41^. The genome and gene sequences were downloaded from Phytozome http://www.phytozome.net (release v1.6 of *Physcomitrella patens*). InterPro database^42^ was used to extract the B3 DNA binding domain of ABI3 genes (access number IPR003340).

### Identification of novel miRNAs

The method for novel miRNA identification has been described in detail in previous studies of plant miRNAs^43^,^44^. Here, we briefly discuss the key steps of the method. First, qualified reads from all libraries that mapped to the known moss miRNAs (miRBase release 21) were excluded from the analysis. The remaining reads were then mapped to the *P. patens* reference genome using Bowtie^41^ and neighboring loci were merged if they had reads overlapping one another. The (merged) loci were extended 300-nt on both ends and segments of 250-nt were extracted using a sliding window starting from the 5′-end. Possible secondary structures of each of the segments were found using RNA-fold^45^. Candidate miRNAs were chosen based on four key criteria, including presence of more than 10 reads, existence of hairpin structures, appearance of miRNA* sequences and RNA-RNA duplex structures with ~2-nt 3′ overhangs.

### Identification of siRNAs

The remaining qualified reads from miRNA discovery were used to search for siRNAs by investigating both genomic and cDNA sequences for clusters with 21-nt reads enriched as siRNA-yielding candidates. Specifically, qualified reads from all small-RNA libraries that were aligned to miRNA loci were removed first. The remaining reads were then aligned to genomic and cDNA sequences with Bowtie (version 0.12.7)^41^ allowing no mismatches. Genome-aligned reads were clustered within a window size of 50-nt to form a putative candidate region. Two stringent criteria were applied to those candidate transcripts and regions with mapped reads. First, candidates with less than 10 mapped reads were removed to ensure a sufficient level of expression. Second, the majority (over 70%) of the mapped reads within a candidate transcript must be 21-nt long. These two criteria aimed to filter out false positive candidates due to random RNA degradation or other types of endogenous small RNAs that do not possess the characteristic of 21-nt enrichment. For ta-siRNAs, we further looked for the presence of at least one miRNA binding site on a siRNA-yielding candidate transcript. These criteria were previously adopted in studies of miRNAs in rice and *Arabidopsis*^20^,^46^.

### Identification of targets of miRNAs and siRNAs

We first applied the target prediction tool, TargetFinder^47^, to identify putative targets of miRNAs and siRNAs. As for siRNAs, we modified the scoring for mismatches (i.e. no double penalties in the 2-nd to 7-th nucleotide of a siRNA sequence) in the TargetFinder method because siRNAs may not obey the seed-region rule as miRNAs. We then integrated degradome data (NCBI GEO GSE16367) with the results from (modified) TargetFinder for detecting candidate target sites with evidence from some sequencing reads. Degradome profiling is a high-throughput method for capturing and profiling 5′-ends of uncapped mRNAs that include miRNA and siRNA cleavage products^37^. To increase detection specificity, we increased the binding score of TargetFinder to 8, which was normally set at 4 (see^47^ for detail of the scoring metric).

## Additional Information

**How to cite this article**: Xia, J. *et al*. Endogenous small-noncoding RNAs and potential functions in desiccation tolerance in *Physcomitrella patens*. *Sci. Rep.* **6**, 30118; doi: 10.1038/srep30118 (2016).

## Supplementary Material

Supplementary Information
