# Supplementary material for: Endogenous Small-Noncoding RNAs and Potential Functions in Desiccation Tolerance in Physcomitrella Patens
Source: Sci Rep. 2016 Jul 22;6:30118. doi: 10.1038/srep30118 (PMC4957126; doi:10.1038/srep30118)
Supplement: Supplementary Information [file srep30118-s1.pdf]

Endogenous small-noncoding RNAs and potential functions in desiccation responses in *Physcomitrella patens*

Jing Xia[1,2], Xiaoqin Wang[3,4,5], Pierre-Francois Perroud[4], Yikun He[3], Ralph Quatrano[4] and Weixiong Zhang[2,6,1†]

[1] Institute for Systems Biology, Jianghan University, Wuhan, Hubei 430056, China;

[2] Department of Computer Science and Engineering, [4] Department of Biology, and [6] Department of Genetics, Washington University, St. Louis, MO 63130, USA;

[3] College of Life Sciences, Capital Normal University, Beijing 100048, China.

[5] Key Laboratory of Urban Agriculture (North) of Ministry of Agriculture, Beijing University of Agriculture, Beijing 102206, China

† Correspondence – WZ: weixiong.zhang@wustl.edu.

Supplemental Figure S1. Base pairings between a novel-miR (query) and a MYB gene (target) are shown in the format as in Figure S1. Reads from a degradome profiling aligned to the miRNA cleavage site were highlighted in red.

```
miRNA ID(query)         target       score       range       gene_annotation
novel_miR21             Pp1s173_12V6.1  6        2102-2123   SER/ARG-rich protein 34A

query   3' GGUCCAU-GUCACUGAAGCUCA 5'
            .. ::: ::::::.::::.::
target  5' ATGTGTAGCAGTGATTTCGGGTAGAGTTTCAGCATTTGAATGTTCTTGCGTGGTCATATACCCAAATGTGCAACAAGGATTCT 3'
               GTAGCAGTGATTTCGGGTAGAGTT_2
                      TGATTTCGGGTAGAGTTTCAGCAT_61
                                 AGAGTTTCAGCATTTGAATGTTCT_10
                                    GTTTCAGCATTTGAATGTTCTTGC_1
                                        CAGCATTTGAATGTTCTTGCGTGG_4
                                         AGCATTTGAATGTTCTTGCGTGGT_2
                                           CATTTGAATGTTCTTGCGTGGTCA_3
                                               TGAATGTTCTTGCGTGGTCATATA_2
                                                  ATGTTCTTGCGTGGTCATATACCC_2
                                                      TCTTGCGTGGTCATATACCCAAAT_1
                                                       CTTGCGTGGTCATATACCCAAATG_2
```

Supplemental Figure S2. A long hairpin structure, **hpRNA-1**, gives rise to siRNA species. (**A** and **B**) siRNA reads form multiple RNA duplexes arranged in different phasing patterns along the same stem structure. (**C**) The genomic loci for novel hpRNA and the read distributions across the locus.

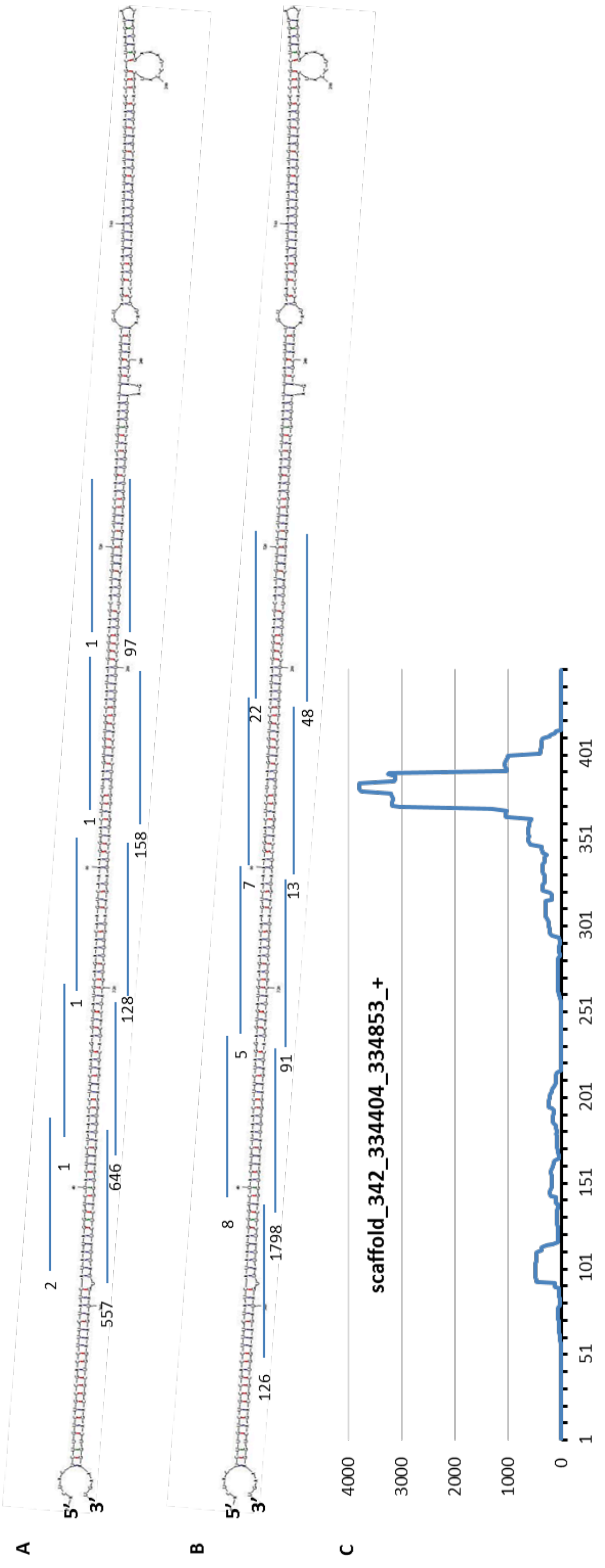

Supplemental Figure S3. A long hairpin structure, **hpRNA-2**, gives rise to siRNA species. The bottom of the diagram displays the read distributions across the genomic loci of the novel hairpin.

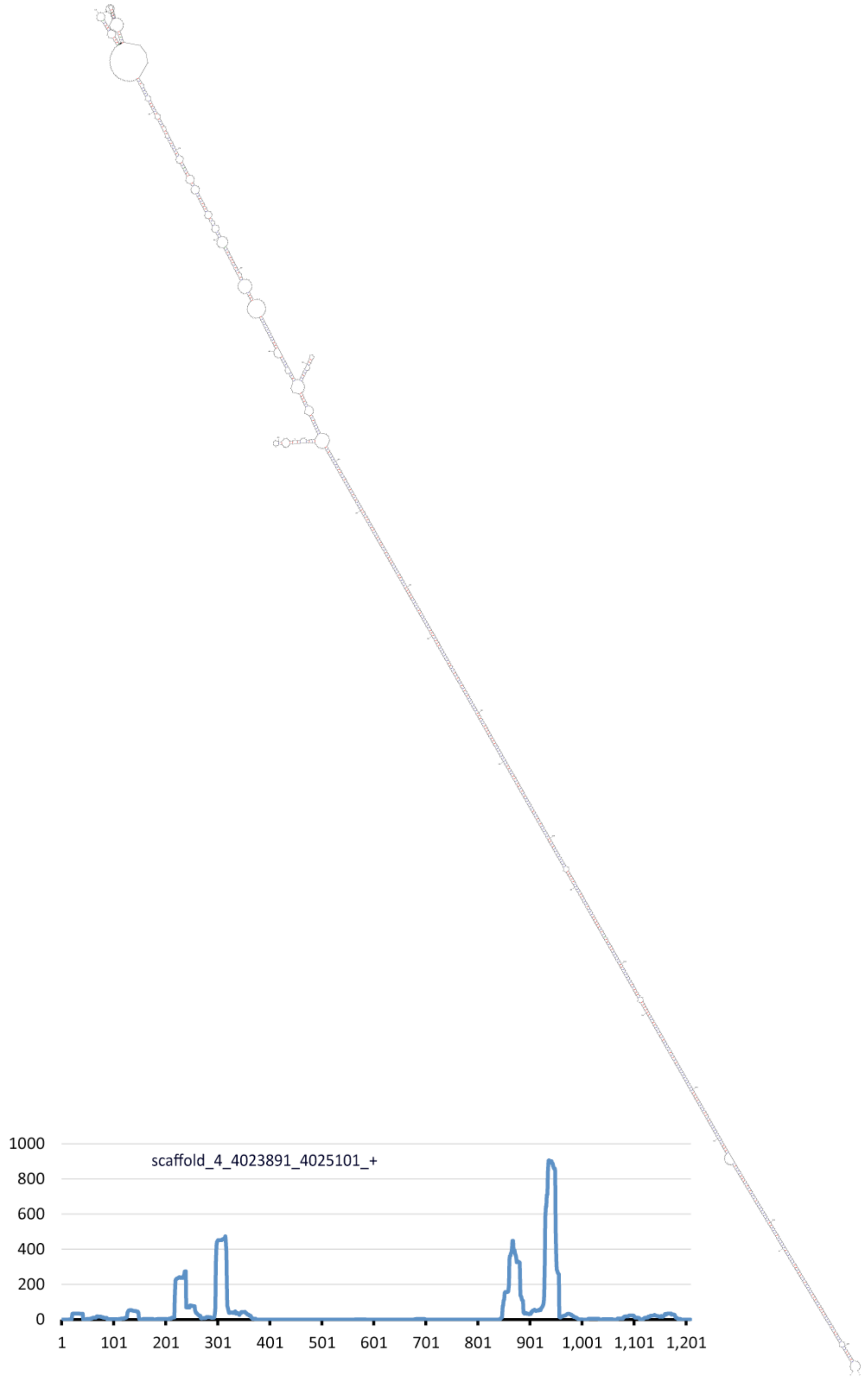

Supplemental Figure S4. Base pairings between the siRNA species from hpRNA-2 (query) and gene Pp1s76_75V6.1 (target) are shown in the format as in Figure S1. Reads from a degradome profiling aligned to the miRNA cleavage site were highlighted in red.

```
siRNA ID(query)                          target      score     range  gene_annotation
hpRNA-2 scaffold_4_4023891_4025101_+_136      Pp1s76_75V6.1   5      209-228 Ribosomal S17 family protein

query   3' CGUCUAGGAAGGUGCUUACUC 5'
           ::: ::: :::::. .: :::
target  5' GCAATCATTCCATCGAAGAGGCTTCGCAATAAGATCGCAGGATTTGCCACTCATTTGATGAAGCGTATCCAGAGGGGACCAGTGCGTGGA 3'
            CAATCATTCCATCGAAGAGGCTTC_1
                  TTCCATCGAAGAGGCTTCGCAATA_3
                   TCCATCGAAGAGGCTTCGCAATAA_1
                      ATCGAAGAGGCTTCGCAATAAGAT_126
                         GAAGAGGCTTCGCAATAAGATCGC_1
                          AAGAGGCTTCGCAATAAGATCGCA_1
                             AGGCTTCGCAATAAGATCGCAGGA_1
                                      AATAAGATCGCAGGATTTGCCACT_3
                                       ATAAGATCGCAGGATTTGCCACTC_2
                                        TAAGATCGCAGGATTTGCCACTCA_3
                                         AAGATCGCAGGATTTGCCACTCAT_5
                                          AGATCGCAGGATTTGCCACTCATT_2
                                           GATCGCAGGATTTGCCACTCATTT_2
                                            ATCGCAGGATTTGCCACTCATTTG_8
                                               GCAGGATTTGCCACTCATTTGATG_3
                                                CAGGATTTGCCACTCATTTGATGA_5
                                                 AGGATTTGCCACTCATTTGATGAA_2
                                                     TTTGCCACTCATTTGATGAAGCGT_2
                                                      TTGCCACTCATTTGATGAAGCGTA_1
                                                       TGCCACTCATTTGATGAAGCGTAT_1
                                                          CACTCATTTGATGAAGCGTATCCA_2
                                                            CTCATTTGATGAAGCGTATCCAGA_1
                                                             TCATTTGATGAAGCGTATCCAGAG_6
                                                              CATTTGATGAAGCGTATCCAGAGG_1
                                                               ATTTGATGAAGCGTATCCAGAGGG_1
                                                                TTTGATGAAGCGTATCCAGAGGGG_3
                                                                 TTGATGAAGCGTATCCAGAGGGGA_4
                                                                  TGATGAAGCGTATCCAGAGGGGAC_3
                                                                   GATGAAGCGTATCCAGAGGGGACC_1
                                                                    ATGAAGCGTATCCAGAGGGGACCA_6
                                                                     TGAAGCGTATCCAGAGGGGACCAG_1
                                                                       AAGCGTATCCAGAGGGGACCAGTG_1
```

Supplemental Table S1. Statistics of raw sequence reads from eight small-RNA libraries from normal (**WT**) and mutant (**ABI3**) *P. patens* under four conditions.

sampleID	condition	raw	qualified	mapped (genome & cDNA)	mapped (miRNA)	miRNA %
sample 1	WT 7d	22,023,071	11,805,438	6,492,651	354614	5.5%
sample 2	WT ABA treatment 24h	23,888,512	15,487,870	9,631,114	228469	2.4%
sample 3	WT after ABA treatment 24h and then dehydrate 24h	22,582,754	13,606,137	9,355,373	178119	1.9%
sample 4	WT after dehydrate 24h and then rehydrate 30min	28,620,792	11,210,278	6,878,694	156251	2.3%
sample 5	ABI3 7d	20,941,360	18,563,669	12,722,857	860992	6.8%
sample 6	ABI3 ABA treatment 24h	23,228,605	13,710,072	8,459,668	382485	4.5%
sample 7	ABI3 after ABA treatment 24h and then dehydrate 24h	22,602,785	14,770,464	9,916,971	262228	2.6%
sample 8	ABI3 after dehydrate 24h and then rehydrate 30min	26,835,253	9,113,175	6,440,788	243738	3.8%

Supplemental Table S2. List of novels miRNAs identified in the current study. Included in the table are miRNA temporary ID, the genomic scaffold ID, the start and end positions, strand (strd.), mature miRNA sequences and the number of qualified reads from all small RNA libraries (#. reads).

temp ID	scaffold ID	start	end	strd.	miRNA on 5p arm	#. Reads	miRNA on 3p arm	#. Reads
novel_miR1	scaffold_137	475811	475970	+	CCATGGCTCTGGTGGTTCTTC	4	AGAACCACCAGAGCCATGGGG	30
novel_miR2	scaffold_143	180165	180324	+	CCCTGGATCAATGCATTGGAA	23	CCAATGCATTGATCCAGGGTG	40
novel_miR3	scaffold_15	2756714	2756873	+	CTCATGAGTGATGGTATGTGC	78	ACATATCATCACTCAAGAGAG	1
novel_miR4	scaffold_165	688983	689142	+	CCTTTTGATGCATTAAATGA	1	CATTTAATGCATCAAAAGGAC	12
novel_miR5	scaffold_20	103704	103863	+	TAGAAGTAATCCGTAGCGAAG	12	TCGCTACAGATCACTTCTATA	3
novel_miR6	scaffold_34	800366	800525	+	TGAAAGCACCCAGAAAATGAT	5	CATTTTCTGGGTGCTTTCATC	2
novel_miR7	scaffold_5	3307718	3307877	+	TGTGATGATAGAAGTTGGAATATA	22	TATTCCAAATTTTATCATCACAAT	1
novel_miR8	scaffold_150	632159	632318	+	AAGTTGATTGATTTGGCACA	4	TGCCAAAATCAATCAACTCGAC	8
novel_miR9	scaffold_77	1413128	1413287	+	TCGCCGACTAGAATTTTATCT	8	ATAAAATTCTAGTCGGCGAAG	5
novel_miR10	scaffold_150	594035	594194	-	TCTTTCTCACACACACTGACA	11	TCAGTGTGTGGGTGTGAGAGC	15
novel_miR11	scaffold_83	1586908	1587067	-	TGGATAAGCGCTCAAGAAACAC	61	TTTCTGAAGCGTTTTTCCATC	83
novel_miR12	scaffold_81	496623	496872	+	ACTTGCTGCTATTTGTGGATG	41	TCCACAAATAGCAGCAAGTGC	3269
novel_miR13	scaffold_99	649008	649257	+	ACAATCTATCACAGTACTTAG	1178	AAGTACTGTGATAGATTGTTG	233
novel_miR14	scaffold_104	412737	412886	+	AGCCATATGTGTAAATCTGTG	88	CAGATTTACACATATGGCTGG	524
novel_miR15	scaffold_79	576842	576991	-	TAAACAATCACAGATCACATT	23	TGTGATCTGTGATTGTTTAAA	115
novel_miR16	scaffold_45	1376161	1376310	-	TGTGGTTGGAATTGTTAGAGC	146	TCTAATAATTCCAACTACAGG	1
novel_miR17	scaffold_46	1285893	1286042	-	TCGGATCGGATTTATGCTTGT	155	ACAAGCACTAATCCGATCCGAC	1
novel_miR18	scaffold_83	1142106	1142255	-	TTACCCCAGTCAAACTATTCC	8	TATAGATTGACTGGGGTAACT	725
novel_miR19	scaffold_375	169687	169786	+	ATGGATTGCCTAAGAGAGACG	401	TCTCTCTTAGCCAATCATGC	2
novel_miR20	scaffold_309	301356	301605	+	CATCTCACTACCATATCCATT	4	TGGACATGGTAGTGAGAAGCT	334
novel_miR21	scaffold_66	898001	898160	+	AGGTACAGAGGCTTCGAGTGG	1	ACTCGAAGTCACTGTACCTGG	26
novel_miR22	scaffold_170	61513	61655	-	TCAAAGATCTCCGTCTGCCCT	1134	GCAGGTGGCGATCTTTGAAGC	2208

Supplemental Table S3. List of known miRNAs in *P. patens*. miRNA ID: numbers after miRNA names indicate the number of individual members producing the same mature miRNA. Included in the table are the number of normalized reads for each condition.

	WT				ABI3 mutant			
**miRNA ID**	**7 day**	**ABA treatment 24h**	**dehydrate 24h**	**rehydrate 30min**	**7 day**	**ABA treatment 24h**	**dehydrate 24h**	**rehydrate 30min**
ppt-MIR319d	80741.6	34018.8	38389.2	105130.0	96780.6	20151.2	20201.4	132643.7
ppt-MIR535d	103970.0	31454.1	25767.1	34935.4	113304.2	62954.2	56132.9	90586.5
ppt-MIR535a	99982.7	29750.4	25190.9	34244.4	110248.9	59506.6	53996.4	88006.3
ppt-MIR535b	99463.2	29725.0	25177.8	34239.3	109363.0	59463.2	53983.2	87972.4
ppt-MIR535c	99433.6	29723.2	25176.9	34238.0	109348.5	59434.3	53978.8	87972.4
ppt-MIR319c	62968.7	31226.4	37037.8	104743.9	87711.5	18765.2	19103.6	132109.2
ppt-MIR319e	62181.5	30917.1	36656.8	103976.7	86605.2	18326.3	18850.8	131375.3
ppt-MIR1028b	79931.5	20065.3	24614.7	8366.8	113619.4	48334.7	28798.6	14878.7
ppt-MIR1028c	13964.5	10722.1	7801.1	1535.7	71122.0	70331.5	30273.5	5191.5
ppt-MIR904a	14478.6	46703.1	3144.5	5078.2	10825.7	80043.1	10023.6	3805.1
ppt-MIR319a	30055.2	9628.0	8674.3	9012.0	47641.7	11220.5	10587.5	16100.9
ppt-MIR904b	23797.6	24286.4	2010.8	2738.5	18113.4	48114.7	8828.0	3468.7
ppt-MIR319b	26502.5	8876.0	7930.0	8525.5	42546.1	9781.8	9836.8	14870.5
ppt-MIR156c	32480.2	8378.8	5887.5	2240.6	19478.6	13463.8	31764.2	9542.0
ppt-MIR156a	32482.9	8392.4	5895.0	2243.2	19453.9	13422.5	31748.3	9537.9
ppt-MIR534a	32055.0	11650.2	4849.9	2457.8	32039.1	17139.6	11032.4	6270.0
ppt-MIR538c	20476.4	12287.0	4726.6	1493.7	21117.9	20505.5	10003.3	3425.3
ppt-MIR538b	19542.5	11830.7	4474.5	1260.0	19929.8	19954.0	9465.0	2710.4
ppt-MIR156b	21489.7	2993.7	3508.8	1340.1	13979.9	8681.8	16856.1	4568.9
ppt-MIR536e	14871.5	3881.0	2583.3	3664.5	17651.9	9832.4	4550.6	7193.8
ppt-MIR1023a	10440.1	2595.5	1361.7	226.1	14577.4	15037.8	7304.7	1608.9
ppt-MIR538a	9603.0	9201.6	3479.8	1260.0	9622.6	10366.4	6866.8	2551.7
ppt-MIR1025	4280.7	6021.0	3240.7	5273.8	6907.2	10989.1	6242.2	9186.6
ppt-MIR536d	10690.4	2567.4	1943.5	3580.7	12274.7	7264.8	3535.6	7271.1
ppt-MIR536b	9784.7	2375.9	1716.6	3489.2	11204.8	6195.9	3099.5	7010.7
ppt-MIR536a	9773.9	2373.2	1716.6	3484.1	11162.9	6176.2	3097.7	7002.5
ppt-MIR1024a	3747.8	1619.3	1708.2	5367.8	9948.8	2600.6	2518.9	16659.8
ppt-MIR1024b	3747.8	1618.4	1711.9	5352.6	9912.4	2599.6	2517.1	16606.9
ppt-MIR894	5783.9	5269.0	2943.7	1126.7	6153.2	2438.5	2777.9	862.8
ppt-MIR1218	4275.3	1458.8	937.7	2838.9	5907.3	3713.0	2012.3	4712.7

ppt-MIR1215	9885.6	617.8	1863.2	603.3	5431.4	1265.2	1936.5	1166.6
ppt-MIR902b	4648.1	1132.2	1346.7	243.9	4047.6	6278.5	2217.6	698.6
ppt-MIR900	3581.0	2829.5	1908.0	656.7	3369.8	4452.5	2077.5	1245.3
ppt-MIR902a	4607.7	1117.7	1329.0	109.2	3926.1	6208.2	2167.4	147.9
ppt-MIR898a	3757.2	1713.7	2433.8	971.7	4084.0	2358.9	1159.5	2117.6
ppt-MIR1212	2898.7	2819.6	2145.2	1187.6	1370.7	2941.5	2094.2	1694.3
ppt-MIR898b	3474.6	1720.0	2060.2	879.0	3724.2	1677.3	988.5	1842.2
ppt-MIR160e	5310.2	1170.3	650.9	276.9	4014.0	2404.4	1248.4	329.6
ppt-MIR1026b	8081.0	381.9	1145.9	463.6	1430.5	465.8	439.6	522.3
ppt-MIR1026a	8068.9	378.3	1145.9	459.8	1423.6	463.7	439.6	516.8
ppt-MIR1221	2785.6	350.2	241.9	246.4	5132.7	625.9	402.6	709.5
ppt-MIR1048	1781.7	539.8	851.7	908.2	1561.6	1291.0	777.1	1820.5
ppt-MIR1052	765.7	991.6	672.4	363.3	1112.5	2560.3	1400.0	1003.8
ppt-MIR529b	1133.1	1437.0	492.2	618.6	1023.9	1882.8	649.3	1064.9
ppt-MIR529c	1126.4	1430.6	493.1	617.3	997.1	1888.0	638.8	1058.1
ppt-MIR1214	1107.5	1402.5	848.9	1004.7	1068.6	1021.5	891.6	879.0
ppt-MIR477c	2695.5	1329.9	612.7	284.5	1272.5	990.5	421.1	207.6
ppt-MIR529a	1040.2	1359.9	429.6	621.1	892.1	1706.2	600.9	1097.5
ppt-MIR1219b	5565.9	90.7	316.6	196.9	984.8	38.2	96.9	202.1
ppt-MIR1065	950.1	805.6	1089.0	514.4	723.8	1449.0	1274.9	596.9
ppt-MIR1217	2037.4	288.5	341.8	393.8	1820.5	1102.0	416.7	625.4
ppt-MIR533c	1793.8	450.0	756.5	243.9	607.8	1183.6	455.5	367.6
ppt-MIR1216	1082.0	507.1	632.3	809.1	754.7	387.3	270.5	1277.9
ppt-MIR1027a	580.0	419.1	363.3	445.8	539.1	492.7	475.8	1033.7
ppt-MIR1027b	580.0	419.1	363.3	445.8	539.1	492.7	475.8	1033.7
ppt-MIR533a	1102.1	334.8	537.0	202.0	429.2	928.5	321.6	352.7
ppt-MIR902e	690.4	56.2	94.3	113.0	1628.9	713.7	423.8	446.3
ppt-MIR902g	690.4	56.2	94.3	111.8	1628.9	713.7	423.8	446.3
ppt-MIR390a	651.3	563.4	423.1	202.0	693.6	900.6	396.5	309.3
ppt-MIR902c	687.7	56.2	90.6	108.0	1624.1	707.5	422.9	438.2
ppt-MIR477f	297.4	957.1	487.5	108.0	206.7	1428.4	423.8	116.7
ppt-MIR390c	977.0	408.2	293.3	53.3	1211.4	674.4	290.7	90.9
ppt-MIR533d	997.2	300.3	535.1	195.6	386.6	778.7	288.1	341.9
ppt-MIR2083	873.4	460.9	386.6	513.2	583.7	130.1	100.4	470.7
ppt-MIR477g	877.4	557.0	214.8	58.4	533.6	787.0	208.8	86.8
ppt-MIR1219a	1258.2	125.2	168.1	247.7	1060.3	84.7	74.0	226.5

ppt-MIR1063d	729.4	222.3	156.9	212.1	857.7	473.0	224.7	219.8
ppt-MIR166l	378.1	616.9	380.1	94.0	519.2	657.9	226.4	202.1
ppt-MIR537b	374.1	208.7	192.4	195.6	445.0	503.0	449.3	694.6
ppt-MIR537c	374.1	208.7	192.4	195.6	445.0	503.0	449.3	694.6
ppt-MIR1078	629.8	275.8	349.3	80.0	560.4	684.8	297.8	130.2
ppt-MIR537d	602.9	158.8	141.0	188.0	588.5	318.1	219.4	624.0
ppt-MIR902h	146.7	174.2	193.3	459.8	315.9	326.4	271.4	721.7
ppt-MIR537a	547.7	155.1	122.3	143.5	534.3	305.7	212.3	552.1
ppt-MIR477e	1041.6	191.4	234.4	232.4	318.6	269.6	86.3	175.0
ppt-MIR1032	339.1	475.4	211.1	78.8	346.1	667.2	314.5	84.1
ppt-MIR166e	390.3	313.9	122.3	100.3	674.4	390.4	124.2	367.6
ppt-MIR166b	394.3	315.7	122.3	99.1	673.7	388.3	124.2	359.5
ppt-MIR901	522.1	229.5	324.1	113.0	382.5	402.8	190.3	165.5
ppt-MIR902i	103.6	136.1	144.8	425.5	259.6	248.9	236.1	656.6
ppt-MIR477a	973.0	157.9	190.5	141.0	307.0	227.2	55.5	131.6
ppt-MIR1063g	530.2	161.5	147.6	224.8	497.2	229.3	124.2	251.0
ppt-MIR477d	889.5	160.6	186.8	128.3	277.4	232.4	43.2	115.3
ppt-MIR529d	395.6	274.0	167.2	71.1	401.7	446.2	146.3	112.6
ppt-MIR477b	876.1	145.2	179.3	175.3	262.3	212.8	43.2	109.9
ppt-MIR1211	285.3	444.5	186.8	151.2	211.5	361.5	139.2	183.1
ppt-MIR1063e	528.9	133.4	137.3	224.8	421.7	162.2	99.6	245.5
ppt-MIR390b	294.7	277.6	211.1	174.0	259.6	234.4	130.4	284.9
ppt-MIR899	662.1	108.9	145.7	208.3	333.8	74.4	51.1	208.9
ppt-MIR171a	94.2	72.6	43.9	376.0	182.0	72.3	43.2	820.7
ppt-MIR166c	179.0	257.6	100.9	99.1	430.6	160.1	88.1	354.1
ppt-MIR1031b	304.1	92.5	123.3	141.0	239.7	213.8	184.1	363.6
ppt-MIR166d	174.9	254.9	100.9	99.1	422.3	160.1	88.1	354.1
ppt-MIR1023b	184.4	53.5	71.0	24.1	540.5	428.6	202.6	149.2
ppt-MIR166h	168.2	254.0	100.9	100.3	418.2	160.1	86.3	363.6
ppt-MIR166a	166.9	254.0	99.9	99.1	415.5	160.1	86.3	354.1
ppt-MIR166g	166.9	254.0	99.9	99.1	415.5	160.1	86.3	354.1
ppt-MIR166i	166.9	253.1	99.9	99.1	414.1	158.0	86.3	355.4
ppt-MIR166f	161.5	246.8	98.1	96.5	404.5	152.9	83.7	335.1
ppt-MIR536c	86.1	137.9	52.3	391.2	87.9	178.7	96.9	533.1
ppt-MIR171b	51.1	66.2	39.2	368.4	144.2	49.6	35.2	807.1
ppt-MIR1060	227.4	117.9	53.2	62.2	344.7	302.6	105.7	47.5

ppt-MIR1035	394.3	83.5	157.8	20.3	321.4	157.0	73.1	51.5
ppt-MIR1222a	441.4	4.5	13.1	34.3	631.8	40.3	22.0	32.6
ppt-MIR1039	858.6	41.7	96.2	19.1	100.3	15.5	15.0	19.0
ppt-MIR1063f	218.0	102.5	100.9	193.1	177.9	95.0	60.8	208.9
ppt-MIR1063b	215.3	103.4	99.9	188.0	164.1	94.0	59.0	198.1
ppt-MIR1033b	366.0	74.4	88.7	87.6	125.0	99.2	75.8	202.1
ppt-MIR1063c	207.2	99.8	99.0	186.7	160.0	91.9	57.3	198.1
ppt-MIR1033c	358.0	76.2	85.0	87.6	125.0	93.0	74.9	199.4
ppt-MIR1063a	200.5	98.9	99.9	182.9	155.9	91.9	58.1	199.4
ppt-MIR1063h	199.2	100.7	99.0	185.4	155.2	89.9	57.3	198.1
ppt-MIR902j	475.0	132.4	248.4	54.6	46.0	57.8	28.2	19.0
ppt-MIR1029	168.2	213.2	46.7	58.4	179.2	201.4	74.0	103.1
ppt-MIR1049	219.4	87.1	66.3	76.2	298.7	114.6	45.8	130.2
ppt-MIR1033d	327.0	66.2	78.5	81.3	116.7	84.7	71.4	191.3
ppt-MIR1033a	328.4	64.4	78.5	81.3	115.4	84.7	71.4	191.3
ppt-MIR1033e	323.0	64.4	78.5	81.3	114.0	84.7	71.4	191.3
ppt-MIR1219c	138.6	79.8	90.6	182.9	257.5	30.0	28.2	185.8
ppt-MIR1059	137.3	168.7	84.1	90.2	147.0	186.9	76.7	85.5
ppt-MIR1074	230.1	127.0	96.2	36.8	179.2	148.7	89.0	51.5
ppt-MIR1031a	121.1	30.8	51.4	144.8	110.6	104.3	56.4	336.4
ppt-MIR1076	57.9	58.1	47.6	168.9	125.0	35.1	34.4	424.6
ppt-MIR1030f	262.4	144.2	87.8	38.1	130.5	163.2	67.8	35.3
ppt-MIR2079	191.1	95.3	48.6	15.2	189.5	215.9	85.5	44.8
ppt-MIR166k	91.5	164.2	71.0	78.8	104.4	86.8	35.2	170.9
ppt-MIR1223c	224.7	84.4	63.5	72.4	173.1	45.4	35.2	73.3
ppt-MIR166j	60.6	165.1	74.7	78.8	96.1	80.6	36.1	170.9
ppt-MIR536f	239.5	23.6	21.5	24.1	147.0	148.7	75.8	77.3
ppt-MIR1223a	205.9	55.3	88.7	116.9	154.5	24.8	29.1	78.7
ppt-MIR529f	55.2	169.6	111.1	36.8	80.3	205.5	44.1	32.6
ppt-MIR902f	47.1	8.2	13.1	77.5	111.9	53.7	44.1	355.4
ppt-MIR1223i	51.1	59.9	61.6	213.4	7.6	63.0	44.1	206.2
ppt-MIR160f	180.3	38.1	23.3	35.6	184.7	123.9	33.5	85.5
ppt-MIR160a	179.0	38.1	23.3	35.6	183.4	123.9	34.4	85.5
ppt-MIR2082	65.9	43.5	52.3	92.7	102.3	68.2	59.9	200.8
ppt-MIR902d	29.6	10.0	10.3	73.7	111.3	49.6	40.5	341.9
ppt-MIR897	110.3	76.2	79.4	6.4	128.4	158.0	80.2	13.6

ppt-MIR1223h	39.0	50.8	45.8	202.0	5.5	56.8	45.8	202.1
ppt-MIR893	113.0	85.3	58.8	27.9	74.9	140.5	59.9	33.9
ppt-MIR1050	26.9	67.1	57.9	82.6	50.1	93.0	44.9	169.6
ppt-MIR1056	49.8	29.9	32.7	78.8	65.2	47.5	22.9	240.1
ppt-MIR1223d	86.1	42.6	30.8	69.9	74.9	43.4	53.7	149.2
ppt-MIR1030d	172.3	64.4	41.1	34.3	111.3	47.5	45.8	29.8
ppt-MIR1222d	265.1	22.7	21.5	26.7	133.9	41.3	14.1	19.0
ppt-MIR1223e	52.5	47.2	76.6	149.9	16.5	42.3	21.1	126.2
ppt-MIR1030b	157.4	67.1	37.4	35.6	103.0	42.3	45.8	28.5
ppt-MIR1030c	118.4	67.1	51.4	54.6	85.8	35.1	43.2	55.6
ppt-MIR1223g	41.7	38.1	60.7	160.0	15.8	26.9	18.5	131.6
ppt-MIR477h	185.7	35.4	40.2	35.6	64.6	39.2	16.7	69.2
ppt-MIR2080	92.9	26.3	29.9	64.8	61.1	68.2	45.8	96.3
ppt-MIR160d	292.0	1.8	0.9	1.3	165.5	4.1	1.8	2.7
ppt-MIR1223f	39.0	36.3	59.8	149.9	13.7	26.9	17.6	122.1
ppt-MIR529e	6.7	138.8	76.6	33.0	1.4	140.5	26.4	27.1
ppt-MIR1030g	107.7	68.0	39.2	36.8	77.6	44.4	41.4	25.8
ppt-MIR1058	39.0	71.7	23.3	45.7	49.4	50.6	81.1	65.1
ppt-MIR1030a	110.3	62.6	34.6	35.6	79.0	35.1	38.8	25.8
ppt-MIR1030e	102.3	62.6	34.6	34.3	83.1	35.1	40.5	27.1
ppt-MIR1037	91.5	32.7	26.2	27.9	94.1	56.8	45.8	29.8
ppt-MIR1043	64.6	108.9	89.7	45.7	39.8	16.5	18.5	5.4
ppt-MIR533b	68.6	40.8	20.5	12.7	92.7	44.4	49.3	29.8
ppt-MIR2084	99.6	28.1	25.2	14.0	92.0	62.0	22.0	8.1
ppt-MIR1066	121.1	25.4	46.7	5.1	79.0	46.5	15.9	8.1
ppt-MIR1223b	90.2	22.7	20.5	43.2	79.0	13.4	19.4	58.3
ppt-MIR1219d	36.3	36.3	18.7	40.6	81.0	15.5	10.6	105.8
ppt-MIR1054	53.8	39.0	43.9	41.9	31.6	57.8	15.0	57.0
ppt-MIR1040	35.0	31.8	14.9	30.5	30.9	87.8	50.2	46.1
ppt-MIR2085	45.8	30.8	11.2	8.9	70.0	54.7	31.7	19.0
ppt-MIR1046	16.1	11.8	19.6	40.6	39.8	21.7	10.6	92.2
ppt-MIR2081	39.0	23.6	22.4	3.8	37.8	46.5	44.1	20.3
ppt-MIR160i	49.8	1.8	1.9	19.1	54.3	4.1	4.4	58.3
ppt-MIR1036	37.7	28.1	12.1	12.7	21.3	30.0	13.2	25.8
ppt-MIR1068	43.1	1.8	7.5	20.3	25.4	14.5	4.4	47.5
ppt-MIR1223j	12.1	0.9	9.3	30.5	7.6	9.3	6.2	62.4

ppt-MIR902k	18.8	11.8	16.8	25.4	20.6	13.4	10.6	20.3
ppt-MIR166m	12.1	19.1	11.2	11.4	12.4	12.4	9.7	39.3
ppt-MIR902l	39.0	6.4	9.3	22.9	17.9	5.2	5.3	16.3
ppt-MIR1220a	36.3	13.6	17.7	29.2	5.5	4.1	0.9	13.6
ppt-MIR533e	39.0	13.6	17.7	5.1	9.6	25.8	6.2	0.0
ppt-MIR1220b	32.3	14.5	8.4	31.8	5.5	2.1	0.0	9.5
ppt-MIR160b	36.3	1.8	3.7	3.8	39.1	4.1	2.6	9.5
ppt-MIR2078	9.4	12.7	8.4	2.5	19.2	14.5	13.2	13.6
ppt-MIR1070	13.5	10.0	5.6	5.1	18.5	13.4	10.6	12.2
ppt-MIR160h	40.4	0.0	1.9	0.0	35.7	0.0	0.0	0.0
ppt-MIR2077	0.0	13.6	20.5	21.6	0.7	8.3	2.6	9.5
ppt-MIR1030h	10.8	11.8	3.7	5.1	11.7	3.1	6.2	6.8
ppt-MIR1047	8.1	2.7	1.9	6.4	11.7	6.2	1.8	19.0
ppt-MIR534b	18.8	9.1	5.6	7.6	8.2	4.1	0.9	1.4
ppt-MIR529g	9.4	1.8	4.7	17.8	4.1	4.1	2.6	10.9
ppt-MIR1057	4.0	2.7	0.9	8.9	0.7	6.2	1.8	28.5
ppt-MIR1038	10.8	1.8	3.7	8.9	7.6	7.2	0.0	12.2
ppt-MIR1222c	2.7	6.4	0.9	3.8	8.9	10.3	7.9	8.1
ppt-MIR1044	2.7	7.3	1.9	6.4	4.1	13.4	5.3	6.8
ppt-MIR1062	6.7	4.5	2.8	2.5	2.1	6.2	5.3	13.6
ppt-MIR1073	0.0	1.8	0.0	1.3	11.0	5.2	4.4	4.1
ppt-MIR1051	2.7	1.8	0.0	1.3	6.9	4.1	1.8	8.1
ppt-MIR419	6.7	4.5	4.7	1.3	2.7	3.1	0.9	0.0
ppt-MIR1075	1.3	0.0	1.9	1.3	11.0	3.1	0.0	4.1
ppt-MIR1061	2.7	1.8	6.5	0.0	4.1	5.2	1.8	0.0
ppt-MIR1053	9.4	0.9	2.8	1.3	2.7	0.0	0.9	2.7
ppt-MIR408b	5.4	5.4	0.0	0.0	4.8	1.0	0.9	1.4
ppt-MIR1055	1.3	0.9	0.9	0.0	8.9	3.1	0.9	2.7
ppt-MIR1030i	4.0	2.7	0.0	0.0	3.4	4.1	0.0	2.7
ppt-MIR895	0.0	4.5	0.9	0.0	4.1	3.1	2.6	0.0

Supplemental Table S4. Differentially expressed sncRNAs in *P. patens* between condition 1 and condition 2. The first column lists the two conditions in a comparison.

Cond1 vs Cond2	miRNA or siRNA ID	Fold change (log2)	cond 1	cond 2
Rehy_Dehy	ppt-miR1028b-3p_1	-1.84	40882.9	146475.1
Rehy_Dehy	ppt-miR319c_3	1.24	512937.5	217685.9
Rehy_Dehy	ppt-miR1028c-3p_1	-2.63	7488	46255.6
Rehy_Dehy	TAS3a_55086	-1.24	22009.5	52038.2
Rehy_Dehy	ppt-miR156a_3	-1.62	6700.8	20548.1
Rehy_Dehy	ppt-miR1023a-3p_1	-3.24	723.2	6832.5
Rehy_Dehy	ppt-miR1024a_2	1.39	26764.6	10206.7
Rehy_Dehy	ppt-miR904a-3p_1	1.23	23718.2	10144.9
Rehy_Dehy	ppt-miR538a_3	-1.94	4723.2	18122.7
Rehy_Dehy	ppt-miR903_1	-4.57	1920	45694.2
Rehy_Dehy	ppt-miR1218_1	1.49	13715.1	4867.5
Rehy_Dehy	ppt-miR1215_1	-1.90	2950.4	11020.7
Rehy_Dehy	ppt-miR902a-3p_2	-3.93	518.4	7904.8
Rehy_Dehy	TAS3a_24606	-4.46	1254.4	27650.1
Rehy_Dehy	ppt-miR904a-5p_2	-2.40	1548.8	8202.4
Rehy_Dehy	ppt-miR898a-5p_2	-1.49	3513.6	9875.4
Rehy_Dehy	ppt-miR1221-5p_1	-1.72	211.2	696.2
Rehy_Dehy	ppt-miR900-5p_1	-1.48	2118.4	5917.4
Rehy_Dehy	ppt-miR1217-5p_1	-1.40	614.4	1628.1
Rehy_Dehy	ppt-miR902c-3p_3	-2.17	108.8	494.1
Rehy_Dehy	ppt-miR1026a_2	-1.57	2304	6832.5
Rehy_Dehy	ppt-miR1212_1	-1.10	5926.4	12733.1
Rehy_Dehy	TAS3a_4121	-1.84	1318.4	4727.2
Rehy_Dehy	novel_miR22-3p	-5.35	25.6	1083.5
Rehy_Dehy	ppt-miR1052_1	-1.25	1612.8	3840.1
Rehy_Dehy	ppt-miR390c-3p_1	-3.72	115.2	1532.7
Rehy_Dehy	TAS3a_325	-2.49	1081.6	6085.8
Rehy_Dehy	ppt-miR1028c-5p_1	-1.32	166.4	415.5
Rehy_Dehy	ppt-miR1065_1	-1.21	2508.8	5805.1
Rehy_Dehy	ppt-miR1023a-5p_1	-1.50	358.4	1016.2
Rehy_Dehy	ppt-miR1078_1	-2.43	384	2071.6

Rehy_Dehy	TAS3c_2545	-1.42	608	1633.7
Rehy_Dehy	TAS3a_2972	-3.24	524.8	4979.8
Rehy_Dehy	ppt-miR533a-3p_3	-1.68	985.6	3149.6
Rehy_Dehy	hpRNA-1	-4.58	38.4	943.2
Rehy_Dehy	ppt-miR529d_1	-1.05	313.6	651.3
Rehy_Dehy	ppt-miR1032_1	-1.64	307.2	960
Rehy_Dehy	TAS3c_1043	-1.92	89.6	342.5
Rehy_Dehy	TAS3a_208	1.03	652.8	320
Rehy_Dehy	novel_miR13-5p	-3.05	51.2	432.3
Rehy_Dehy	ppt-miR1217-3p_1	1.83	1318.4	370.5
Rehy_Dehy	novel_miR13-3p	-2.27	140.8	684.9
Rehy_Dehy	novel_miR22-5p	2.31	780.8	157.2
Rehy_Dehy	TAS3c_arf	-1.65	217.6	684.9
Rehy_Dehy	novel-miR12-3p	4.10	4652.8	269.5
Rehy_Dehy	ppt-miR536c_1	4.19	1760	95.4

Supplemental Table S5. Prediticion of miRNA binding sites on ABA-induced genes. Scores are reported by TargetFinder. Range indicates the start and end of binding sites residing on cDNAs.

miRNA	gene ID	gene symbol	score	range	alignment		
ppt-miR477h	Pp1s7_115V6.1	PpABI3A	2.5	64-82	target 5' UCGGAGCCUUUGAGGGAGC 3'	: :.::::::::::::::	query 3' ACCUUCGGAAACUCCCUCU 5'
ppt-miR1038-3p	Pp1s65_246V6.1	sorbitol_dehyd	4.5	729-749	target 5' UCUGGCUGCGAUUCCACCAAU 3'	.:::: :::::::::::::	query 3' GGACCUACGCUAAGGUGGUAC 5'
ppt-miR1220a	Pp1s67_7V6.1	porin	4.5	1199-1220	target 5' CUGUCUUUCCUCGCCACCUGAA 3'	::.:: ::::::.::::: :::	query 3' GAUAG-AAGGAGUGGUGGCCUU 5'
ppt-miR1220b	Pp1s67_7V6.1	porin	4.5	1199-1220	target 5' CUGUCUUUCCUCGCCACCUGAA 3'	::.:: ::::::.::::: :::	query 3' GAUAG-AAGGAGUGGUGGCCUU 5'
ppt-miR477a-5p	Pp1s7_115V6.1	PpABI3A	4.5	64-83	target 5' UCGGAGCCUUUGAGGGAGCC 3'	: :.::::::::::::::	query 3' ACCUUCGGAAACUCCCUCUC 5'
ppt-miR477b	Pp1s7_115V6.1	PpABI3A	4.5	64-83	target 5' UCGGAGCCUUUGAGGGAGCC 3'	: :.::::::::::::::	query 3' ACCUUCGGAAACUCCCUCUC 5'
ppt-miR477c	Pp1s7_115V6.1	PpABI3A	4.5	64-83	target 5' UCGGAGCCUUUGAGGGAGCC 3'	: :.::::::::::::::	query 3' ACCUUCGGAAACUCCCUCUC 5'
ppt-miR477d	Pp1s7_115V6.1	PpABI3A	4.5	64-83	target 5' UCGGAGCCUUUGAGGGAGCC 3'	: :.::::::::::::::	query 3' ACCUUCGGAAACUCCCUCUC 5'
ppt-miR477e	Pp1s7_115V6.1	PpABI3A	4.5	64-83	target 5' UCGGAGCCUUUGAGGGAGCC 3'	: :.::::::::::::::	query 3' ACCUUCGGAAACUCCCUCUC 5'
ppt-miR477f	Pp1s7_115V6.1	PpABI3A	4.5	60-80	target 5' GAGGUCGGAGCCUUUGAGGGA 3'	: : :.:::::::::::::	query 3' AACAACCUUCGGAAACUCCCU 5'
ppt-miR477g-5p	Pp1s7_115V6.1	PpABI3A	4.5	60-80	target 5' GAGGUCGGAGCCUUUGAGGGA 3'	: : :.:::::::::::::	query 3' AACAACCUUCGGAAACUCCCU 5'
ppt-miR156a	Pp1s381_18V6.2	PpACT5	5	148-166	target 5' UUGCAUACUCUCUUCUG-CG 3'	::: .:::::::::: :.	query 3' CACGAGUGAGAGAAGACAGU 5'
ppt-miR156b	Pp1s381_18V6.2	PpACT5	5	148-166	target 5' UUGCAUACUCUCUUCUG-CG 3'	::: .:::::::::: :.	query 3' CACGAGUGAGAGAAGACAGU 5'
ppt-miR319a	Pp1s66_200V6.1	MYB	5	924-943	target 5' GGAGCUCCCUUCAUGCCAAU 3'	:::::::::::: ::::	query 3' CCUCGAGGGAAGUCAGGUUC 5'
ppt-miR156c	Pp1s381_18V6.2	PpACT5	5	148-166	target 5' UUGCAUACUCUCUUCUG-CG 3'	::: .:::::::::: :.	query 3' CACGAGUGAGAGAAGACAGU 5'
ppt-miR536f	Pp1s7_115V6.1	PpABI3A	5	1617-1636	target 5' UGCACACAGCUUGGCUUGUA 3'	::::::::::::::: .: :	query 3' ACGUGUGUCGAACCGUGCUU 5'
ppt-miR536f	Pp1s89_139V6.2	sut4	5	131-150	target 5' UGCGCGCAGCUUGGCCUGGA 3'	:::.:.::::::::: .:.:	query 3' ACGUGUGUCGAACCGUGCUU 5'
novel_miR14-3p	Pp1s379_42V6.2	lea_group3	5	1278-1298	target 5' UCGCUCGUCUGUGUAAAUCUG 3'	.:. .:.: :::::::::::	query 3' GGUCGGUAUACACAUUUAGAC 5'
novel_miR15-3p	Pp1s67_7V6.1	porin	5.5	1603-1622	target 5' UUUAAACGUUCACAG-UCGCA 3'	:::::::. :::::: ::.::	query 3' AAAUUUGUUAGUGUCUAGUGU 5'
ppt-miR536f	Pp1s173_143V6.1	PpABI3B	5.5	1233-1252	target 5' CGCAUAUAGCUUGGCGAGAG 3'	:::.:.::::::::. ::.	query 3' ACGUGUGUCGAACCGUGCUU 5'
ppt-miR902a-3p	Pp1s234_91V6.2	lea_group3	5.5	420-439	target 5' GAUGGGACGCAGACCUUCGA 3'	: :. :: ::::::::::	query 3' CGAUACUACGUCUGGAAGCA 5'
ppt-miR319a	Pp1s143_30V6.1	MYB	5.5	1422-1441	target 5' GGGGAUCCCUUCGGACCAAA 3'	::.: :::::::.: ::::	query 3' CCUCGAGGGAAGUCAGGUUC 5'
ppt-miR319a	Pp1s391_54V6.1	MYB	5.5	1496-1515	target 5' GGGGAUCCCUUCGGACCAAA 3'	::.: :::::::.: ::::	query 3' CCUCGAGGGAAGUCAGGUUC 5'
ppt-miR1037	Pp1s10_147V6.1	Phypa_159190	5.5	1225-1244	target 5' UUGU-AAAUCCUAAUAAAGUU 3'	....: ::::::::::: :.:	query 3' GGUAGUUUAGGAUUAUUCCGA 5'
ppt-miR1046-5p	Pp1s6_369V6.1	Phypa_113212	5.5	1042-1060	target 5' CCUGAGAAG-AUGAAAUCCU 3'	: :::.::. :::::::::	query 3' GCACUUUUUAUACUUUAGGU 5'
ppt-miR1217-3p	Pp1s10_147V6.1	Phypa_159190	5.5	1261-1280	target 5' CUUAAUA-CAGGUUUCAAAUU 3'	::: :.: :: :.::::::::	query 3' GAACUGUAGUACGAAGUUUAA 5'
ppt-miR477g-3p	Pp1s213_122V6.1	Phypa_194258	5.5	624-643	target 5' UUUUCCAU-AAGGUUUCCAAA 3'	:.:.:::. ::::.::::::	query 3' AGAGGGUGCUUCCGAAGGUUG 5'
ppt-miR536a	Pp1s89_139V6.2	sut4	6	128-150	target 5' GUCUGCGCGCAGCUUGGCCUGGA 3'	:: :::.:.::::::::: .:.:	query 3' CA-ACGUGUGUCGAACCGUGCUU 5'
ppt-miR536b	Pp1s89_139V6.2	sut4	6	128-150	target 5' GUCUGCGCGCAGCUUGGCCUGGA 3'	:: :::.:.::::::::: .:.:	query 3' CA-ACGUGUGUCGAACCGUGCUU 5'
ppt-miR536d	Pp1s89_139V6.2	sut4	6	128-150	target 5' GUCUGCGCGCAGCUUGGCCUGGA 3'	:: :::.:.::::::::: .:.:	query 3' CA-ACGUGUGUCGAACCGUGCUU 5'
ppt-miR536e	Pp1s89_139V6.2	sut4	6	128-150	target 5' GUCUGCGCGCAGCUUGGCCUGGA 3'	:: :::.:.::::::::: .:.:	query 3' CA-ACGUGUGUCGAACCGUGCUU 5'
ppt-miR536f	Pp1s379_42V6.2	lea_group3	6	1212-1232	target 5' AGCUUAUAGUUGUGGCACGAA 3'	:: .:.::.: :::::::::	query 3' ACGUGUGUCGA-ACCGUGCUU 5'
ppt-miR902a-5p	Pp1s7_115V6.1	PpABI3A	6	640-656	target 5' UUAGGAA-CUGCAGCAUG 3'	: :.::: ::::: :::.	query 3' ACUUCUUAGACGUAGUAU 5'
ppt-miR902c-3p	Pp1s234_91V6.2	lea_group3	6	420-439	target 5' GAUGGGACGCAGACCUUCGA 3'	. :. :: ::::::::::	query 3' UGAUACUACGUCUGGAAGCA 5'
ppt-miR902e-3p	Pp1s234_91V6.2	lea_group3	6	420-439	target 5' GAUGGGACGCAGACCUUCGA 3'	. :. :: ::::::::::	query 3' UGAUACUACGUCUGGAAGCA 5'
ppt-miR902g-3p	Pp1s234_91V6.2	lea_group3	6	420-439	target 5' GAUGGGACGCAGACCUUCGA 3'	. :. :: ::::::::::	query 3' UGAUACUACGUCUGGAAGCA 5'
ppt-miR1045	Pp1s143_30V6.1	MYB	6	1603-1623	target 5' CGCGUCGAAGUGCCAGCACUA 3'	.::::.::. :::::::::	query 3' UUGCAGUUUUUCGGUCGUGAA 5'
ppt-miR1221-5p	Pp1s202_106V6.1	Phypa_169276	6	74-94	target 5' UUUGGGCUCGCGCACCGUCCA 3'	::::. :. ::.::::.::::	query 3' AAACUGGGACGUGUGGUAGGU 5'
novel_miR6	Pp1s67_7V6.1	porin	6.5	242-261	target 5' UUCGUUGU-UGGGUGCUUGCA 3'	::.:: : ::::::::: ::	query 3' UAGUAAAAGACCCACGAAAGU 5'
ppt-miR160a	Pp1s213_122V6.1	Phypa_194258	6.5	534-554	target 5' UUUUAUGUGGGCAGCCAGGCA 3'	: .::...:: :::::::::	query 3' ACCGUAUGUCCCUCGGUCCGU 5'
ppt-miR160e	Pp1s213_122V6.1	Phypa_194258	6.5	534-554	target 5' UUUUAUGUGGGCAGCCAGGCA 3'	: .::...:: :::::::::	query 3' ACCGUAUGUCCCUCGGUCCGU 5'
ppt-miR160f	Pp1s213_122V6.1	Phypa_194258	6.5	534-554	target 5' UUUUAUGUGGGCAGCCAGGCA 3'	: .::...:: :::::::::	query 3' ACCGUAUGUCCCUCGGUCCGU 5'
ppt-miR477a-5p	Pp1s10_147V6.1	Phypa_159190	6.5	1124-1143	target 5' GGGAAGUCUUUUGUGGAGAG 3'	:::::.:::: . ::::::	query 3' ACCUUCGGAAACUCCCUCUC 5'
ppt-miR477b	Pp1s10_147V6.1	Phypa_159190	6.5	1124-1143	target 5' GGGAAGUCUUUUGUGGAGAG 3'	:::::.:::: . ::::::	query 3' ACCUUCGGAAACUCCCUCUC 5'
ppt-miR477c	Pp1s10_147V6.1	Phypa_159190	6.5	1124-1143	target 5' GGGAAGUCUUUUGUGGAGAG 3'	:::::.:::: . ::::::	query 3' ACCUUCGGAAACUCCCUCUC 5'
ppt-miR477d	Pp1s10_147V6.1	Phypa_159190	6.5	1124-1143	target 5' GGGAAGUCUUUUGUGGAGAG 3'	:::::.:::: . ::::::	query 3' ACCUUCGGAAACUCCCUCUC 5'
ppt-miR477e	Pp1s10_147V6.1	Phypa_159190	6.5	1124-1143	target 5' GGGAAGUCUUUUGUGGAGAG 3'	:::::.:::: . ::::::	query 3' ACCUUCGGAAACUCCCUCUC 5'
ppt-miR533b-5p	Pp1s6_369V6.1	Phypa_113212	6.5	746-765	target 5' AUCUC-CAGCCUGAGCAGCUG 3'	.::: ::::::: .:::::	query 3' GGGAGUGUCGGACCUGUCGAG 5'
ppt-miR902c-3p	Pp1s202_106V6.1	Phypa_169276	6.5	493-511	target 5' AUU-UGAAGCAGAACUUCGA 3'	:.: ::: ::::: :::::	query 3' UGAUACUACGUCUGGAAGCA 5'
ppt-miR902e-3p	Pp1s202_106V6.1	Phypa_169276	6.5	493-511	target 5' AUU-UGAAGCAGAACUUCGA 3'	:.: ::: ::::: :::::	query 3' UGAUACUACGUCUGGAAGCA 5'
ppt-miR902g-3p	Pp1s202_106V6.1	Phypa_169276	6.5	493-511	target 5' AUU-UGAAGCAGAACUUCGA 3'	:.: ::: ::::: :::::	query 3' UGAUACUACGUCUGGAAGCA 5'
ppt-miR1036-5p	Pp1s3_7V6.1	PM_protein	6.5	627-646	target 5' CA-CCGAUUACGGCCACCACA 3'	:: : .::::::: : :::::	query 3' GUCGAUUAAUGCCUGAGGUGU 5'
ppt-miR1058	Pp1s65_246V6.1	sorbitol_dehyd	6.5	1846-1866	target 5' GUGCAUUGUGAUGAGAUCUUC 3'	:::: :.:::::: .::::.	query 3' CACGAAGCACUACCUUAGAGA 5'
ppt-miR1219d	Pp1s67_175V6.1	Pp_specific_4	6.5	159-178	target 5' CAGUU-GUGAGAGGAAAGAAA 3'	::.: :::::::: : ::::	query 3' UUCGAUCACUCUCCGUCCUUU 5'
ppt-miR1222b	Pp1s143_82V6.1	PpABI3C	6.5	846-866	target 5' UCUUUAAAUGGAAUCCUUCAG 3'	: : . ::::.: ::::::::	query 3' ACAUGGUUACUUGAGGAAGUC 5'
ppt-miR1222c	Pp1s143_82V6.1	PpABI3C	6.5	846-866	target 5' UCUUUAAAUGGAAUCCUUCAG 3'	: : . ::::.: ::::::::	query 3' ACAUGGUUACUUGAGGAAGUC 5'
ppt-miR2082	Pp1s143_82V6.1	PpABI3C	6.5	1494-1515	target 5' AAGAAAGAAGCAGAGCAGCACA 3'	::. ::::::: ::.:: ::::	query 3' UUUCUUCUUCGCCUUGU-GUGU 5'

ppt-miR390c-3p	Pp1s381_18V6.2	PpACT5	6.5	983-1002	target	5' CGCUGAUCGGGAUGGA-AGCG 3'	:. :: ::.:.:::: ::::	query	3' GUUACGAGUCUUACCUGUCGC 5'
ppt-miR390c-3p	Pp1s381_21V6.1	PpACT5	6.5	982-1001	target	5' CGCUGAUCGGGAUGGA-AGCG 3'	:. :: ::.:.:::: ::::	query	3' GUUACGAGUCUUACCUGUCGC 5'
ppt-miR477h	Pp1s75_99V6.1	lea_group3	6.5	280-297	target	5' UGGCAGCC-UUGAGGGGCG 3'	::: :::: :::::::. .	query	3' ACCUUCGGAAACUCCCUCU 5'
ppt-miR536c	Pp1s173_143V6.1	PpABI3B	6.5	1231-1252	target	5' AACGCAUAUAGCUUGGCGAGAG 3'	:: :::.: ::::::::. ::	query	3' UUUCGUGUUUCGAACCGUGCUA 5'
ppt-miR536f	Pp1s143_82V6.1	PpABI3C	6.5	1781-1800	target	5' UGCACACAGCUCGGCUAGAG 3'	:::::::::: ::: ::.	query	3' ACGUGUGUCGAACCGUGCUU 5'
ppt-miR902d-3p	Pp1s234_91V6.2	lea_group3	6.5	420-439	target	5' GAUGGGACGCAGACCUUCGA 3'	: :. :: :::::::::.	query	3' CGAUGCUACGUCUGGAAGUA 5'
ppt-miR1033a	Pp1s114_139V6.1	lea_group3	7	701-720	target	5' AGU-CUCACCACGACGUGUCG 3'	::: :.:: :::::: .:::.	query	3' UCACGGGUAGUGCUGGGCAGU 5'
ppt-miR1033b	Pp1s114_139V6.1	lea_group3	7	701-720	target	5' AGU-CUCACCACGACGUGUCG 3'	::: :.:: :::::: .:::.	query	3' UCACGGGUAGUGCUGGGCAGU 5'
ppt-miR1033c	Pp1s114_139V6.1	lea_group3	7	701-720	target	5' AGU-CUCACCACGACGUGUCG 3'	::: :.:: :::::: .:::.	query	3' UCACGGGUAGUGCUGGGCAGU 5'
ppt-miR1033d	Pp1s114_139V6.1	lea_group3	7	701-720	target	5' AGU-CUCACCACGACGUGUCG 3'	::: :.:: :::::: .:::.	query	3' UCACGGGUAGUGCUGGGCAGU 5'
ppt-miR1033e	Pp1s114_139V6.1	lea_group3	7	701-720	target	5' AGU-CUCACCACGACGUGUCG 3'	::: :.:: :::::: .:::.	query	3' UCACGGGUAGUGCUGGGCAGU 5'
ppt-miR1048-5p	Pp1s353_22V6.1	Pp_specific_2	7	23-44	target	5' GUUGCUUACACUCUUGUUUAUA 3'	::.: .::::::: ::::. ::	query	3' CAGCAGAUGUGAGUACAAG-AU 5'
ppt-miR1059	Pp1s7_115V6.1	PpABI3A	7	839-860	target	5' GUAGUGUAUCGGAGGAUUUUCA 3'	:: :: : : :.::::.:::::	query	3' CAACAAACA-CUUCCUGAAAGU 5'
ppt-miR1073-3p	Pp1s143_30V6.1	MYB	7	440-460	target	5' CGUCAAAUGUACUAGCGCCUG 3'	.: ::::::: ::.:::::..	query	3' AUACUUUACAUAAUUGCGGGU 5'
ppt-miR1076-3p	Pp1s67_7V6.1	porin	7	1073-1094	target	5' CGGUUUGAUGAUAAGGGUUUGG 3'	:.: :: .:::::: :.::::	query	3' GUCCCAC-GCUAUUCACGAACC 5'
ppt-miR1212	Pp1s353_22V6.1	Pp_specific_2	7	748-768	target	5' CUCAUCCUGAGCUGUCCUACA 3'	: ::: ::. ::::::::.::	query	3' GCGUAAGAUACGACAGGGUGC 5'
ppt-miR1215	Pp1s353_22V6.1	Pp_specific_2	7	36-57	target	5' UUGUUUAUAGUUUAGCAAGUGU 3'	:.:: ::.::::: :::: ::	query	3' AGCAUAUGUCAAAACGUU-ACU 5'
ppt-miR1222a	Pp1s143_82V6.1	PpABI3C	7	846-866	target	5' UCUUUAAAUGGAAUCCUUCAG 3'	: : . ::::.: ::::::::.	query	3' AUAUGGUUACUUGAGGAAGUU 5'
ppt-miR2080	Pp1s33_341V6.1	eif4	7	1425-1444	target	5' CCAUUUGUGAAGUGGU-UGGA 3'	:::.::.::: ::.: ::::	query	3' CGUAGACGCUUAACUAUACCU 5'
ppt-miR2082	Pp1s379_42V6.2	lea_group3	7	516-536	target	5' GAAGGAGAAGCUGAACGAAUA 3'	.:::.:::::: ::::. :.:	query	3' UUUCUUCUUCGCCUUGUGUGU 5'
ppt-miR408b	Pp1s114_139V6.1	lea_group3	7	1608-1628	target	5' AGCCAAGGAAGACGCAGGUCA 3'	::::: :::::: :::: ::	query	3' UCGGUCCCUUCUCCGUCACGU 5'
ppt-miR477h	Pp1s169_138V6.1	pyr_dehyd	7	282-301	target	5' GGGGGGCUUUUGAAGGGAAA 3'	::..::.:::: ::::: :	query	3' ACCUUCGGAAAC-UCCCUCU 5'
ppt-miR533b-5p	Pp1s34_349V6.1	ppg4	7	238-257	target	5' UCCAC-UAGCCUGGACAUGUC 3'	.:: : .::::::::: ::	query	3' GGGAGUGUCGGACCUGUCGAG 5'
ppt-miR533e	Pp1s75_99V6.1	lea_group3	7	1520-1541	target	5' GGGGACCAAGCAGACUGGUGGC 3'	:.:. ::: ::::::: :::.	query	3' CUCUCGGUACGUCUGA-CACUC 5'
ppt-miR536a	Pp1s379_42V6.2	lea_group3	7	1210-1232	target	5' GCAGCUUAUAGUUGUGGCACGAA 3'	: :: .:.::.: :::::::::	query	3' CAACGUGUGUCGA-ACCGUGCUU 5'
ppt-miR536a	Pp1s7_115V6.1	PpABI3A	7	1615-1636	target	5' ACUGCACACAGCUUGGCUUGUA 3'	::::::::::::::: .: :	query	3' CAACGUGUGUCGAACCGUGCUU 5'
ppt-miR536b	Pp1s379_42V6.2	lea_group3	7	1210-1232	target	5' GCAGCUUAUAGUUGUGGCACGAA 3'	: :: .:.::.: :::::::::	query	3' CAACGUGUGUCGA-ACCGUGCUU 5'
ppt-miR536b	Pp1s7_115V6.1	PpABI3A	7	1615-1636	target	5' ACUGCACACAGCUUGGCUUGUA 3'	::::::::::::::: .: :	query	3' CAACGUGUGUCGAACCGUGCUU 5'
ppt-miR536d	Pp1s379_42V6.2	lea_group3	7	1210-1232	target	5' GCAGCUUAUAGUUGUGGCACGAA 3'	: :: .:.::.: :::::::::	query	3' CAACGUGUGUCGA-ACCGUGCUU 5'
ppt-miR536d	Pp1s7_115V6.1	PpABI3A	7	1615-1636	target	5' ACUGCACACAGCUUGGCUUGUA 3'	::::::::::::::: .: :	query	3' CAACGUGUGUCGAACCGUGCUU 5'
ppt-miR536e	Pp1s379_42V6.2	lea_group3	7	1210-1232	target	5' GCAGCUUAUAGUUGUGGCACGAA 3'	: :: .:.::.: :::::::::	query	3' CAACGUGUGUCGA-ACCGUGCUU 5'
ppt-miR536e	Pp1s7_115V6.1	PpABI3A	7	1615-1636	target	5' ACUGCACACAGCUUGGCUUGUA 3'	::::::::::::::: .: :	query	3' CAACGUGUGUCGAACCGUGCUU 5'
ppt-miR893	Pp1s143_30V6.1	MYB	7	1612-1631	target	5' GUGCCAGCACUAGUUCUCAU 3'	:: ::::::: :::.:. ::	query	3' CAGGGUCGUGUUCAGGGUUA 5'
ppt-miR902a-3p	Pp1s10_181V6.1	elipa	7	365-383	target	5' GC-AUCACGUGGGCCUUCGU 3'	:: :: : :..:.:::::::	query	3' CGAUACUACGUCUGGAAGCA 5'
ppt-miR902a-3p	Pp1s67_175V6.1	Pp_specific_4	7	1010-1028	target	5' CGAAUGAUGUAGA-CUUUGU 3'	::::::.::: :::.::	query	3' CGAUACUACGUCUGGAAGCA 5'
ppt-miR902b-3p	Pp1s10_181V6.1	elipa	7	365-383	target	5' GC-AUCACGUGGGCCUUCGU 3'	:: :: : :..:.:::::::	query	3' CGAUACUACGUCUGGAAGCA 5'
ppt-miR902b-3p	Pp1s67_175V6.1	Pp_specific_4	7	1010-1028	target	5' CGAAUGAUGUAGA-CUUUGU 3'	::::::.::: :::.::	query	3' CGAUACUACGUCUGGAAGCA 5'
ppt-miR902c-3p	Pp1s67_175V6.1	Pp_specific_4	7	1010-1028	target	5' CGAAUGAUGUAGA-CUUUGU 3'	::::::.::: :::.::	query	3' UGAUACUACGUCUGGAAGCA 5'
ppt-miR902e-3p	Pp1s67_175V6.1	Pp_specific_4	7	1010-1028	target	5' CGAAUGAUGUAGA-CUUUGU 3'	::::::.::: :::.::	query	3' UGAUACUACGUCUGGAAGCA 5'
ppt-miR902f-3p	Pp1s169_138V6.1	pyr_dehyd	7	144-162	target	5' GUUA-GUUGCACAGCUUUGU 3'	:::: : :::: : :::.::	query	3' CAAUACUACGUCUAGAAGCA 5'
ppt-miR902f-3p	Pp1s67_175V6.1	Pp_specific_4	7	1010-1028	target	5' CGAAUGAUGUAGA-CUUUGU 3'	::::::.::: :::.::	query	3' CAAUACUACGUCUAGAAGCA 5'
ppt-miR902g-3p	Pp1s67_175V6.1	Pp_specific_4	7	1010-1028	target	5' CGAAUGAUGUAGA-CUUUGU 3'	::::::.::: :::.::	query	3' UGAUACUACGUCUGGAAGCA 5'
ppt-miR902h-3p	Pp1s143_82V6.1	PpABI3C	7	2579-2598	target	5' GCUU-UGAUGUAGAGUUUUCC 3'	: :: ::::::::: ..:::	query	3' CAAAUACUACAUCUGGGAAGA 5'
ppt-miR902k-5p	Pp1s7_115V6.1	PpABI3A	7	637-656	target	5' ACCUUAGGAA-CUGCAGCAUG 3'	: : :.::: :::::.:::.	query	3' UUUACUUCUUAGACGUUGUAU 5'
ppt-miR902l-5p	Pp1s7_115V6.1	PpABI3A	7	637-656	target	5' ACCUUAGGAA-CUGCAGCAUG 3'	: : :.::: :::::.:::.	query	3' UUUACUUCUUAGACGUUGUAU 5'
ppt-miR903	Pp1s379_42V6.2	lea_group3	7	646-668	target	5' GCUCAUGGUGCUGCUGAUGUAGC 3'	:::: : :: :.::.:: :::::	query	3' CGAG-AACAGGGCGGCUUCAUCG 5'
ppt-miR1046-5p	Pp1s213_122V6.1	Phypa_194258	7	1467-1485	target	5' UGU-AUAGAUAUGAUAUCUG 3'	.:: : :.:::::: :::..	query	3' GCACUUUUUAUACUUUAGGU 5'
ppt-miR1215	Pp1s4_10V6.1	Phypa_112415	7	488-508	target	5' UUGUACAUGGUUAUGCGAUGU 3'	:.::: :..::: :::.:::	query	3' AGCAUAUGUCAAAACGUUACU 5'
ppt-miR1219d	Pp1s10_147V6.1	Phypa_159190	7	785-804	target	5' AAGCU-GUGAGUGCCAAGAAA 3'	::::: ::::: : :: ::::	query	3' UUCGAUCACUCUCCGUCCUUU 5'
ppt-miR477h	Pp1s10_147V6.1	Phypa_159190	7	1124-1142	target	5' GGGAAGUCUUUUGUGGAGA 3'	:::::.:::: . :::::	query	3' ACCUUCGGAAACUCCCUCU 5'
ppt-miR536f	Pp1s213_122V6.1	Phypa_194258	7	1356-1376	target	5' UGUUUGAAGGCUUGGCAGGAA 3'	::. . : .:::::::: :::	query	3' ACGUG-UGUCGAACCGUGCUU 5'
novel_miR6	Pp1s114_139V6.1	lea_group3	7	113-133	target	5' AUUGUGUUGUUGGUGUUUUCA 3'	::..: :: : ::::.:::::	query	3' UAGUAAAAGACCCACGAAAGU 5'
novel_miR14-3p	Pp1s381_21V6.1	PpACT5	7.5	1736-1756	target	5' CCUGUAAUAUUUGUAUAUUUG 3'	:: :. :::: :::: ::.::	query	3' GGUCGGUAUACACAUUUAGAC 5'
novel_miR10	Pp1s211_95V6.1	rehydrin	7.5	109-129	target	5' UUUCGGUGUGUCGGAGAGAGU 3'	: ::.::::: ::::.::	query	3' ACAGUCACACACACUCUUUCU 5'
novel_miR6	Pp1s143_82V6.1	PpABI3C	7.5	273-294	target	5' AUAGUUGUUUUGGGUGGAUUCA 3'	:: .:: ::.:::::: ::::	query	3' UAGUAA-AAGACCCACGAAAGU 5'
novel_miR16	Pp1s173_143V6.1	PpABI3B	7.5	512-532	target	5' GCCCGAGAAGUCCCAACCAUA 3'	:: : :. :.: :::::::.:	query	3' CGAGAUUGUUAAGGUUGGUGU 5'
novel_miR17-5p	Pp1s33_341V6.1	eif4	7.5	1617-1636	target	5' ACAAGUA-GAAUCCGACUUGU 3'	:::::.: .::::::: ..:	query	3' UGUUCGUAUUUAGGCUAGGCU 5'
novel_miR17-5p	Pp1s379_42V6.2	lea_group3	7.5	157-177	target	5' ACAGCCAUGAGUCCGCUUCGU 3'	:::. :::.:.:::: :.::	query	3' UGUUCGUAUUUAGGCUAGGCU 5'
ppt-miR1031a	Pp1s42_233V6.1	Phypa_122983	7.5	464-484	target	5' GGGCGAUCAAGAGAGCUUUGG 3'	.:. : :: ::::::: :::.	query	3' UCUUCGAGGUCUCUCGUAACU 5'

ppt-miR1031b	Pp1s42_233V6.1	Phypa_122983	7.5	464-484	target 5' GGGCGAUCAAGAGAGCUUUGG 3'	.:. : :: ::::::: :::.	query 3' UCUUCGAGGUCUCUCGUAACU 5'
ppt-miR1052	Pp1s42_233V6.1	Phypa_122983	7.5	243-264	target 5' UACCACCACCAAGGACAAGGAG 3'	:::::: : ::: .: :::::.	query 3' AUGGUGUUAGUUAUU-UUCCUU 5'
ppt-miR1053-5p	Pp1s10_147V6.1	Phypa_159190	7.5	973-995	target 5' AGGUCCACGUUGAGA-AACAUAUA 3'	:.:::::: :::::: ::: .::	query 3' UUCAGGUGAAACUCUAUUGGGUAG 5'
ppt-miR1220a	Pp1s202_106V6.1	Phypa_169276	7.5	153-174	target 5' CUCGUCGUGGUCGCCGCCGGAA 3'	:: .:: : ::.::.::::::	query 3' GA-UAGAAGGAGUGGUGGCCUU 5'
ppt-miR1220b	Pp1s202_106V6.1	Phypa_169276	7.5	153-174	target 5' CUCGUCGUGGUCGCCGCCGGAA 3'	:: .:: : ::.::.::::::	query 3' GA-UAGAAGGAGUGGUGGCCUU 5'
ppt-miR319d-5p.1	Pp1s6_369V6.1	Phypa_113212	7.5	230-250	target 5' UAUUGCACCGAACAAGAACUU 3'	::::: :::::: :: : ::.	query 3' AUAACCUGGCUUCUU-UCGAG 5'
ppt-miR893	Pp1s141_128V6.1	Phypa_190133	7.5	559-578	target 5' CUCAAGGCACAGGUCCAGAU 3'	:: .:::::.:::: .::	query 3' CAGGGUCGUGUUCAGGGUUA 5'
ppt-miR893	Pp1s241_56V6.1	Phypa_27775	7.5	549-567	target 5' CUCUC-GCACAAGCUCCCAU 3'	::.: ::::::: .:: ::	query 3' CAGGGUCGUGUUCAGGGUUA 5'
ppt-miR902d-3p	Pp1s213_122V6.1	Phypa_194258	7.5	902-921	target 5' GGUAUCAUGCACACUCUCAU 3'	: ::. ::::: ::. ::::	query 3' CGAUGCUACGUCUGGAAGUA 5'
ppt-miR1023a-3p	Pp1s173_143V6.1	PpABI3B	7.5	956-976	target 5' AGGCGCACUUACCAAUUCUUG 3'	: ::.: ::. ::::::::.	query 3' UACGUGAGAGAGGUUAAGAGA 5'
ppt-miR1027a	Pp1s12_126V6.1	per1	7.5	389-410	target 5' GGUUGGAUUGAGGACAUUGAAA 3'	:.::::: :::.: :: ::::	query 3' CUAACCU-UCUCUUCUAUCUUU 5'
ppt-miR1027b	Pp1s12_126V6.1	per1	7.5	389-410	target 5' GGUUGGAUUGAGGACAUUGAAA 3'	:.::::: :::.: :: ::::	query 3' CUAACCU-UCUCUUCUAUCUUU 5'
ppt-miR1038-3p	Pp1s227_58V6.1	lea_group3	7.5	332-352	target 5' UCUGCAUGGGAAUCCACCAAG 3'	.::: ::: :: ::::::: :	query 3' GGACCUACGCUAAGGUGGUAC 5'
ppt-miR1038-5p	Pp1s196_69V6.1	Pp_specific_3	7.5	930-948	target 5' CGUU-GCGGAGACGAGCCUG 3'	: :: : :::.::: .:::.	query 3' GAAACCACCUUUGCGUGGAU 5'
ppt-miR1043-5p	Pp1s67_175V6.1	Pp_specific_4	7.5	1083-1102	target 5' UGGAUGCUUGAAGGUGAAUG 3'	:: :.:: ::::: :::: :	query 3' ACGUGCGUACUUCAACUUCC 5'
ppt-miR1044-3p	Pp1s114_139V6.1	lea_group3	7.5	1975-1994	target 5' AAAACUGAUGUGUACCCCAA 3'	::::: .::.::.:: :::	query 3' UUUUGUUUAUACGUGAUGUU 5'
ppt-miR1044-3p	Pp1s65_246V6.1	sorbitol_dehyd	7.5	380-399	target 5' AACGCAAACGUGCACCAUAA 3'	:: .:::: .::::: :.::	query 3' UUUUGUUUAUACGUGAUGUU 5'
ppt-miR1047-3p	Pp1s89_139V6.2	sut4	7.5	438-458	target 5' CGUCCAGCCCAUCGUUGGUCA 3'	:. : :: :::::.::.:::	query 3' GUUGAUCCAGUAGCGACUAGU 5'
ppt-miR1049	Pp1s143_82V6.1	PpABI3C	7.5	1661-1681	target 5' ACAUCGUUUGGUUGAAGGAGA 3'	: :. ::::::.:.: .::::	query 3' UCUGACAAACCGAUUCUCUCU 5'
ppt-miR1050	Pp1s34_349V6.1	ppg4	7.5	1013-1033	target 5' CGUUCGGGAGCAAGGUGGACG 3'	: .:::.: :::::::: :.	query 3' UCCGGCCUUAGUUCCACCAGU 5'
ppt-miR1057	Pp1s196_69V6.1	Pp_specific_3	7.5	634-654	target 5' AUCCCCAUGUACAACA-AGGAA 3'	:.::::: :: :::: :.:::	query 3' UGGGGGUCGAUCUUGUGUUCUU 5'
ppt-miR1065	Pp1s7_115V6.1	PpABI3A	7.5	1715-1736	target 5' CUAGCGGUAAGUUAAAGAUUGA 3'	:: :::. ::::.: :::.::	query 3' GA-CGCUCUUCAGUCUCUGACA 5'
ppt-miR1211-5p	Pp1s169_138V6.1	pyr_dehyd	7.5	42-62	target 5' GUUUCAUGCCCCUCUCUCCCU 3'	:: :::. :: ::.::::::	query 3' GAACGUAUUGGUAGGGAGGGA 5'
ppt-miR160a	Pp1s353_22V6.1	Pp_specific_2	7.5	881-900	target 5' UGG-AAGCAGUGAGUUAGGUA 3'	::: : .::: :::..:::.:	query 3' ACCGUAUGUCCCUCGGUCCGU 5'
ppt-miR160e	Pp1s353_22V6.1	Pp_specific_2	7.5	881-900	target 5' UGG-AAGCAGUGAGUUAGGUA 3'	::: : .::: :::..:::.:	query 3' ACCGUAUGUCCCUCGGUCCGU 5'
ppt-miR160f	Pp1s353_22V6.1	Pp_specific_2	7.5	881-900	target 5' UGG-AAGCAGUGAGUUAGGUA 3'	::: : .::: :::..:::.:	query 3' ACCGUAUGUCCCUCGGUCCGU 5'
ppt-miR319d-5p.2	Pp1s234_91V6.2	lea_group3	7.5	426-447	target 5' ACGCAGACCUUCGAUAGAGCCA 3'	:.:: ::::::::. :::::	query 3' CGUGU-UGGAAGCUGAGUCGGU 5'
ppt-miR477a-5p	Pp1s169_138V6.1	pyr_dehyd	7.5	282-302	target 5' GGGGGGCUUUUGAAGGGAAAU 3'	::..::.:::: ::::: :	query 3' ACCUUCGGAAAC-UCCCUCUC 5'
ppt-miR477b	Pp1s169_138V6.1	pyr_dehyd	7.5	282-302	target 5' GGGGGGCUUUUGAAGGGAAAU 3'	::..::.:::: ::::: :	query 3' ACCUUCGGAAAC-UCCCUCUC 5'
ppt-miR477c	Pp1s169_138V6.1	pyr_dehyd	7.5	282-302	target 5' GGGGGGCUUUUGAAGGGAAAU 3'	::..::.:::: ::::: :	query 3' ACCUUCGGAAAC-UCCCUCUC 5'
ppt-miR477d	Pp1s169_138V6.1	pyr_dehyd	7.5	282-302	target 5' GGGGGGCUUUUGAAGGGAAAU 3'	::..::.:::: ::::: :	query 3' ACCUUCGGAAAC-UCCCUCUC 5'
ppt-miR477e	Pp1s169_138V6.1	pyr_dehyd	7.5	282-302	target 5' GGGGGGCUUUUGAAGGGAAAU 3'	::..::.:::: ::::: :	query 3' ACCUUCGGAAAC-UCCCUCUC 5'
ppt-miR477h	Pp1s1_740V6.1	Pp_specific_1	7.5	2-19	target 5' UGGACG-CUUUGAGGCACG 3'	:::: : :::::::: : .	query 3' ACCUUCGGAAACUCCCUCU 5'
ppt-miR533a-5p	Pp1s67_175V6.1	Pp_specific_4	7.5	542-561	target 5' CCUCCACA-CCUUCCCAGCUC 3'	::. :::: ::: :::::::	query 3' GGGAGUGUCGGACCGGUCGAG 5'
ppt-miR536d	Pp1s143_82V6.1	PpABI3C	7.5	1779-1800	target 5' UUUGCACACAGCUCGGCUAGAG 3'	::::::::::: ::: ::.	query 3' CAACGUGUGUCGAACCGUGCUU 5'
ppt-miR536e	Pp1s143_82V6.1	PpABI3C	7.5	1779-1800	target 5' UUUGCACACAGCUCGGCUAGAG 3'	::::::::::: ::: ::.	query 3' CAACGUGUGUCGAACCGUGCUU 5'
ppt-miR893	Pp1s173_143V6.1	PpABI3B	7.5	1138-1156	target 5' GUCCU-UUACAAGAUCCAAG 3'	::::. .::::: .::::	query 3' CAGGGUCGUGUUCAGGGUUA 5'
ppt-miR893	Pp1s32_160V6.1	fqr1	7.5	541-561	target 5' UUAUCAGCACAGGUUCUCAAG 3'	: .::::::::.: ::.:::	query 3' CAGGGUCGUGUUC-AGGGUUA 5'
ppt-miR897	Pp1s227_58V6.1	lea_group3	7.5	71-92	target 5' UGUGCUGCGAUUUUACUUG-UCG 3'	: ::::: : ::..::::: ::.	query 3' AAACGACUCAAAGGUGAACUAGU 5'
ppt-miR898a-3p	Pp1s381_21V6.1	PpACT5	7.5	1319-1339	target 5' UGUCCAUGUGCACAGUCUACC 3'	:.:: ::::::: :.::: :	query 3' AUAGCGACACGUGACGGAUCG 5'
ppt-miR902a-3p	Pp1s169_138V6.1	pyr_dehyd	7.5	144-162	target 5' GUUA-GUUGCACAGCUUUGU 3'	:.:: : :::: : :::.::	query 3' CGAUACUACGUCUGGAAGCA 5'
ppt-miR902a-5p	Pp1s211_95V6.1	rehydrin	7.5	1796-1814	target 5' AGGAGAAGCUGCACCACUA 3'	:.:::: ::::: :: ::	query 3' ACUUCUUAGACGUAGU-AU 5'
ppt-miR902a-5p	Pp1s75_99V6.1	lea_group3	7.5	421-438	target 5' UGGACAAACUGCAACAGA 3'	::.: :: ::::: :: :	query 3' ACUUCUUAGACGUAGUAU 5'
ppt-miR902b-3p	Pp1s169_138V6.1	pyr_dehyd	7.5	144-162	target 5' GUUA-GUUGCACAGCUUUGU 3'	:.:: : :::: : :::.::	query 3' CGAUACUACGUCUGGAAGCA 5'
ppt-miR902c-3p	Pp1s10_181V6.1	elipa	7.5	365-383	target 5' GC-AUCACGUGGGCCUUCGU 3'	.: :: : :..:.:::::::	query 3' UGAUACUACGUCUGGAAGCA 5'
ppt-miR902d-3p	Pp1s65_246V6.1	sorbitol_dehyd	7.5	428-446	target 5' GC-GCGUUGUAGAGUUUCAC 3'	:: .:: ::.::: .::::	query 3' CGAUGCUACGUCUGGAAGUA 5'
ppt-miR902e-3p	Pp1s10_181V6.1	elipa	7.5	365-383	target 5' GC-AUCACGUGGGCCUUCGU 3'	.: :: : :..:.:::::::	query 3' UGAUACUACGUCUGGAAGCA 5'
ppt-miR902g-3p	Pp1s10_181V6.1	elipa	7.5	365-383	target 5' GC-AUCACGUGGGCCUUCGU 3'	.: :: : :..:.:::::::	query 3' UGAUACUACGUCUGGAAGCA 5'
ppt-miR902j-5p	Pp1s7_115V6.1	PpABI3A	7.5	638-657	target 5' CCUUAGGAA-CUGCAGCAUGU 3'	: :.::: :::::.:::.:	query 3' UUACUUCUUAGACGUUGUAUA 5'
ppt-miR902k-5p	Pp1s65_246V6.1	sorbitol_dehyd	7.5	577-596	target 5' GAAUGAAGAGU-GGCGACAUU 3'	.::::::::.: ::.::::	query 3' UUUACUUCUUAGACGUUGUAU 5'
ppt-miR902l-5p	Pp1s65_246V6.1	sorbitol_dehyd	7.5	577-596	target 5' GAAUGAAGAGU-GGCGACAUU 3'	.::::::::.: ::.::::	query 3' UUUACUUCUUAGACGUUGUAU 5'
ppt-miR1025	Pp1s143_82V6.1	PpABI3C	8	2095-2115	target 5' GUUUUUAGGUUUGCUGUGCUA 3'	::: :::: :::: :::: .:	query 3' CAAUAAUCGAAACAACACCGU 5'
ppt-miR1027a	Pp1s118_232V6.1	lea_group3	8	205-225	target 5' GAUUUGGAGAGGAGAAGGCAG 3'	:::: :.::::.::: .: :.	query 3' CUAACCUUCUCUUCUAUCUUU 5'
ppt-miR1027a	Pp1s7_115V6.1	PpABI3A	8	1731-1750	target 5' GAUU-GAAGUGAAGGCAGAGC 3'	:::: :::: ::::. :::.	query 3' CUAACCUUCUCUUCUAUCUUU 5'
ppt-miR1027b	Pp1s118_232V6.1	lea_group3	8	205-225	target 5' GAUUUGGAGAGGAGAAGGCAG 3'	:::: :.::::.::: .: :.	query 3' CUAACCUUCUCUUCUAUCUUU 5'
ppt-miR1027b	Pp1s7_115V6.1	PpABI3A	8	1731-1750	target 5' GAUU-GAAGUGAAGGCAGAGC 3'	:::: :::: ::::. :::.	query 3' CUAACCUUCUCUUCUAUCUUU 5'
ppt-miR1028c-3p	Pp1s353_22V6.1	Pp_specific_2	8	654-675	target 5' GCUGAUGAACCCACAAUGAUCG 3'	::: :.:::: :::::: .:.	query 3' CGAGAAUUUGGAUGUUAC-GGU 5'
ppt-miR1030j	Pp1s65_246V6.1	sorbitol_dehyd	8	1328-1348	target 5' UGUUGUGGGUGCUUAUGCUGG 3'	:: ::..::::: :::: ::	query 3' ACCACGUCCACGUCUACGUCC 5'
ppt-miR1032	Pp1s169_138V6.1	pyr_dehyd	8	1314-1333	target 5' GUCAGAUCCUGGCAG-CACUU 3'	.::. ::: ::::: :::.:	query 3' GGGUUAAGGUCCGUCAGUGGA 5'

ppt-miR1039-5p	Pp1s65_246V6.1	sorbitol_dehyd	8	232-252	target 5' UUGAGGAGAAGAACGCAAAGA 3'	. : .:::::. :: ::::::	query 3' GUCCUCUCUUUCUGGGUUUCU 5'
ppt-miR1041	Pp1s34_349V6.1	ppg4	8	635-655	target 5' AGGACAAGCCGUACCCGAAGA 3'	::::: : : .::::::::.:	query 3' UCCUGGUGAGAGUGGGCUUUU 5'
ppt-miR1043-3p	Pp1s89_139V6.2	sut4	8	806-827	target 5' AGAUUUUCAGUUUCACGCACAC 3'	:: ..::::. ::::::: ::	query 3' UC-GGAAGUUUAAGUGCGCGUU 5'
ppt-miR1048-5p	Pp1s196_69V6.1	Pp_specific_3	8	24-43	target 5' GUCGAAUACACUUG-GUUUUC 3'	:::: ::::::.. :::.:	query 3' CAGCAGAUGUGAGUACAAGAU 5'
ppt-miR1054	Pp1s353_22V6.1	Pp_specific_2	8	278-298	target 5' AAGAAGUGGGUUGAGGGUUUA 3'	:: :.:.:. :::::::::	query 3' GUCCUUAUCUCUCUCCCAAAU 5'
ppt-miR1075	Pp1s196_69V6.1	Pp_specific_3	8	532-551	target 5' GCGGAAAUCCUGACUG-GGCA 3'	: .::::.: :::::: ..::	query 3' CAUCUUUGGUACUGACUUUGU 5'
ppt-miR1217-5p	Pp1s143_82V6.1	PpABI3C	8	2125-2144	target 5' GCGAUUCGCGACUUGA-ACUA 3'	:: ::: ::.:: ::: ::.:	query 3' CGGUAAACGUUGUACUAUGGU 5'
ppt-miR1219a	Pp1s173_143V6.1	PpABI3B	8	272-292	target 5' UAGCUAGUGAGGGGUAUGAUC 3'	::::::::::.::.: ::	query 3' UUCGAUCACUCUCCGUCCUUC 5'
ppt-miR1219b	Pp1s173_143V6.1	PpABI3B	8	272-292	target 5' UAGCUAGUGAGGGGUAUGAUC 3'	::::::::::.::.: ::	query 3' UUCGAUCACUCUCCGUCCUUC 5'
ppt-miR1219c	Pp1s173_143V6.1	PpABI3B	8	272-292	target 5' UAGCUAGUGAGGGGUAUGAUC 3'	::::::::::.::.: ::	query 3' UUCGAUCACUCUCCGUCCUUC 5'
ppt-miR1219d	Pp1s173_143V6.1	PpABI3B	8	272-292	target 5' UAGCUAGUGAGGGGUAUGAUC 3'	::::::::::.::.: ::	query 3' UUCGAUCACUCUCCGUCCUUU 5'
ppt-miR1222d	Pp1s143_82V6.1	PpABI3C	8	2033-2053	target 5' UAUGGCGGUGAACCCCAAGAC 3'	:::. :.:::::::: .:	query 3' AUAUGGUCACUUGGGGAAUUU 5'
ppt-miR160c	Pp1s353_22V6.1	Pp_specific_2	8	881-900	target 5' UGG-AAGCAGUGAGUUAGGUA 3'	::: : :::: :::..:::.	query 3' ACCGUACGUCCCUCGGUCCGC 5'
ppt-miR2078	Pp1s379_42V6.2	lea_group3	8	363-384	target 5' GCAGGAGAAGGCAGGCGACACC 3'	.:::: . :::::.:: : :::	query 3' UGUCCGUGUCCGUUCGGU-UGG 5'
ppt-miR2078	Pp1s75_99V6.1	lea_group3	8	1055-1075	target 5' GCAGGAGAAGGCAGGGCAAGC 3'	.:::: . :::::.: ::: :	query 3' UGUCCGUGUCCGUUCGGUUGG 5'
ppt-miR2082	Pp1s379_42V6.2	lea_group3	8	330-350	target 5' GAAGGAGAAGCUGAACAUCUU 3'	.:::.:::::: :::::. .	query 3' UUUCUUCUUCGCCUUGUGUGU 5'
ppt-miR2083-3p	Pp1s143_82V6.1	PpABI3C	8	828-848	target 5' ACUUCUGAUGCUUUUCAAUCU 3'	::::: ..::. :::::::	query 3' AGAAGAACGUGAGCAGUUAGA 5'
ppt-miR319a	Pp1s7_115V6.1	PpABI3A	8	976-995	target 5' GGUCUUUCCUUCAAUCCUGG 3'	:: .:.:::::: ::: .:	query 3' CCUCGAGGGAAGUCAGGUUC 5'
ppt-miR319b	Pp1s7_115V6.1	PpABI3A	8	976-995	target 5' GGUCUUUCCUUCAAUCCUGG 3'	:: .:.:::::: ::: .:	query 3' CCUCGAGGGAAGUCAGGUUC 5'
ppt-miR390c-3p	Pp1s10_181V6.1	elipa	8	120-140	target 5' CAACUCUCAGCUUGGGCGGCG 3'	::: ::::: :::.:.:::	query 3' GUUACGAGUCUUACCUGUCGC 5'
ppt-miR390c-3p	Pp1s379_42V6.2	lea_group3	8	882-901	target 5' CAAAACCCAGGAUGGAUA-UG 3'	::: : :::.:::::.: .:	query 3' GUUACGAGUCUUACCUGUCGC 5'
ppt-miR408b	Pp1s234_91V6.2	lea_group3	8	443-463	target 5' AGCCAAGGUGGAGGCAGGGGA 3'	::::: :: .::::::: : :	query 3' UCGGUCCCUUCUCCGUCACGU 5'
ppt-miR477a-5p	Pp1s7_115V6.1	PpABI3A	8	1665-1685	target 5' UGAGGAGUCUUUCAAGGAUAG 3'	:: :.::.:::: : ::: ::	query 3' AC-CUUCGGAAACUCCCUCUC 5'
ppt-miR477a-5p	Pp1s75_99V6.1	lea_group3	8	280-298	target 5' UGGCAGCC-UUGAGGGGCGC 3'	::: :::: :::::::. .	query 3' ACCUUCGGAAACUCCCUCUC 5'
ppt-miR477b	Pp1s7_115V6.1	PpABI3A	8	1665-1685	target 5' UGAGGAGUCUUUCAAGGAUAG 3'	:: :.::.:::: : ::: ::	query 3' AC-CUUCGGAAACUCCCUCUC 5'
ppt-miR477b	Pp1s75_99V6.1	lea_group3	8	280-298	target 5' UGGCAGCC-UUGAGGGGCGC 3'	::: :::: :::::::. .	query 3' ACCUUCGGAAACUCCCUCUC 5'
ppt-miR477c	Pp1s7_115V6.1	PpABI3A	8	1665-1685	target 5' UGAGGAGUCUUUCAAGGAUAG 3'	:: :.::.:::: : ::: ::	query 3' AC-CUUCGGAAACUCCCUCUC 5'
ppt-miR477c	Pp1s75_99V6.1	lea_group3	8	280-298	target 5' UGGCAGCC-UUGAGGGGCGC 3'	::: :::: :::::::. .	query 3' ACCUUCGGAAACUCCCUCUC 5'
ppt-miR477d	Pp1s7_115V6.1	PpABI3A	8	1665-1685	target 5' UGAGGAGUCUUUCAAGGAUAG 3'	:: :.::.:::: : ::: ::	query 3' AC-CUUCGGAAACUCCCUCUC 5'
ppt-miR477e	Pp1s75_99V6.1	lea_group3	8	280-298	target 5' UGGCAGCC-UUGAGGGGCGC 3'	::: :::: :::::::. .	query 3' ACCUUCGGAAACUCCCUCUC 5'
ppt-miR477h	Pp1s114_139V6.1	lea_group3	8	751-769	target 5' AAGGAGUCUUCGAAGGAGG 3'	:.::.::: :: ::::.	query 3' ACCUUCGGAAACUCCCUCU 5'
ppt-miR529a	Pp1s65_246V6.1	sorbitol_dehyd	8	2220-2240	target 5' GAGCUCUGCUCUUUAUGUUUG 3'	: ::: ::::::.: : ::.:	query 3' CCCGACACGAGAGAGAGAAGC 5'
ppt-miR529b	Pp1s65_246V6.1	sorbitol_dehyd	8	2220-2240	target 5' GAGCUCUGCUCUUUAUGUUUG 3'	: ::: ::::::.: : ::.:	query 3' CCCGACACGAGAGAGAGAAGC 5'
ppt-miR529c	Pp1s65_246V6.1	sorbitol_dehyd	8	2220-2240	target 5' GAGCUCUGCUCUUUAUGUUUG 3'	: ::: ::::::.: : ::.:	query 3' CCCGACACGAGAGAGAGAAGC 5'
ppt-miR533b-5p	Pp1s7_115V6.1	PpABI3A	8	983-1002	target 5' CCUUCA-AUCCUGGACAGAAU 3'	::.::: : ::::::::: .	query 3' GGGAGUGUCGGACCUGUCGAG 5'
ppt-miR536f	Pp1s33_341V6.1	eif4	8	1105-1124	target 5' UGCGCACAAUUUGGGACGCG 3'	:::.:::: .:::: ::: .	query 3' ACGUGUGUCGAACCGUGCUU 5'
ppt-miR893	Pp1s89_139V6.2	sut4	8	841-861	target 5' GUCGCCUGUGCAAAUCUCAAG 3'	::: :: :..::: ::.:::	query 3' CAG-GGUCGUGUUCAGGGUUA 5'
ppt-miR902a-5p	Pp1s381_18V6.2	PpACT5	8	1058-1075	target 5' GGAAGGAUCUGUAUGGGA 3'	::::.::::::.:: . :	query 3' ACUUCUUAGACGUAGUAU 5'
ppt-miR902a-5p	Pp1s381_21V6.1	PpACT5	8	1057-1074	target 5' GGAAGGAUCUGUAUGGGA 3'	::::.::::::.:: . :	query 3' ACUUCUUAGACGUAGUAU 5'
ppt-miR902c-3p	Pp1s169_138V6.1	pyr_dehyd	8	144-162	target 5' GUUA-GUUGCACAGCUUUGU 3'	..:: : :::: : :::.::	query 3' UGAUACUACGUCUGGAAGCA 5'
ppt-miR902e-3p	Pp1s169_138V6.1	pyr_dehyd	8	144-162	target 5' GUUA-GUUGCACAGCUUUGU 3'	..:: : :::: : :::.::	query 3' UGAUACUACGUCUGGAAGCA 5'
ppt-miR902g-3p	Pp1s169_138V6.1	pyr_dehyd	8	144-162	target 5' GUUA-GUUGCACAGCUUUGU 3'	..:: : :::: : :::.::	query 3' UGAUACUACGUCUGGAAGCA 5'
ppt-miR902h-5p	Pp1s114_139V6.1	lea_group3	8	695-713	target 5' AUGAAGAGUCU-CACCACGA 3'	:::::::.::: :: :: .:	query 3' UACUUCUUAGAUGUAGUAUU 5'
ppt-miR902i-5p	Pp1s114_139V6.1	lea_group3	8	695-713	target 5' AUGAAGAGUCU-CACCACGA 3'	:::::::.::: :: :: .:	query 3' UACUUCUUAGAUGUAGUAUU 5'
novel_miR14-3p	Pp1s143_82V6.1	PpABI3C	8	1570-1589	target 5' CGGGUCAUAUCUG-GAAUCUC 3'	: .:.::::: :: .:::::	query 3' GGUCGGUAUACACAUUUAGAC 5'
novel_miR14-5p	Pp1s32_160V6.1	fqr1	8	398-417	target 5' UGCAGAUUUACCCA-AUGCCG 3'	..::::::::: :: ::: :	query 3' GUGUCUAAAUGUGUAUACCGA 5'
novel_miR14-5p	Pp1s75_99V6.1	lea_group3	8	1911-1932	target 5' AACAAGUUUUGCAUAUGUAGCU 3'	:: :: :::.::.::.: :::	query 3' GUG-UCUAAAUGUGUAUACCGA 5'
novel_miR5	Pp1s118_232V6.1	lea_group3	8	200-220	target 5' CAAGGGAUUUGGAGAGGAGAA 3'	::: :::.:: :: :.::::	query 3' CUUCACUAGACAUCGCUUCUU 5'
novel_miR6	Pp1s65_246V6.1	sorbitol_dehyd	8	1323-1344	target 5' AUCGGUGUUGUGGGUGCUUAUG 3'	::: .: :: ::::::::: ..	query 3' UAG-UAAAAGACCCACGAAAGU 5'
novel_miR7	Pp1s118_232V6.1	lea_group3	8	22-46	target 5' UAUCAUCCAGCUUCUAUUAAUCUCG 3'	::: ::::.:::::::. ::: :.	query 3' AUAUAAGGUUGAAGAUAG-UAGUGU 5'
novel_miR12-5p	Pp1s7_115V6.1	PpABI3A	8	1706-1726	target 5' CAGAAACAGCUAGCGGUAAGU 3'	:: :::. ::::.:.::::	query 3' GUAGGUGUUUAUCGUCGUUCA 5'
novel_miR11	Pp1s353_22V6.1	Pp_specific_2	8	330-351	target 5' GAUGGGAAAUCGGAUUUAGAAG 3'	:::::.::: : : ::.::::.	query 3' CUACCUUUUUG-CGAAGUCUUU 5'
ppt-miR2080	Pp1s241_56V6.1	Phypa_27775	8	115-134	target 5' GCAGC-GAGGGCUGAUGUGGA 3'	::: : : :.. ::::.::::	query 3' CGUAGACGCUUAACUAUACCU 5'
ppt-miR2082	Pp1s6_369V6.1	Phypa_113212	8	541-561	target 5' GAAAAAGAUGCGGUAGACACG 3'	.:: :::: :::: : ::::.	query 3' UUUCUUCUUCGCCUUGUGUGU 5'
ppt-miR902a-5p	Pp1s10_147V6.1	Phypa_159190	8	1073-1090	target 5' UGUCGGAUACGCGUCAUA 3'	:: :.:: ::.:::::	query 3' ACUUCUUAGACGUAGUAU 5'

Supplemental Table S6. Mapping statistics of known TAS Loci in *P. patens*. Included in the table are initiating miRNAs and their binding sites on TAS genes.

		plus strand		minus strand							
**name**	**genomic or cDNA loci**	**#. Reads**	**20-22nt %**	**#. Reads**	**20-22nt %**	**miRNA**	**score**	**range**		**alignment**	
TAS3a	scaffold_61_112817_113633_+	37722	87.0%	121350	97.7%	ppt-miR390	2.5	376-396	target 5' GGCGUUAUCCCUCUUGAGCUG 3'	::::.:::::::::.::::::	query 3' CCGCGAUAGGGAGGACUCGAA 5'
							6	609-630	target 5' GUUGUAUAUCACUCCUGAGCUA 3'	: .:. :::: ::::::::::	query 3' CCGCG-AUAGGGAGGACUCGAA 5'
TAS3b	scaffold_13_2972400_2972613_-	792	92.0%	1211	98.2%	ppt-miR390	4	5-25	target 5' GGCGGUAACCCUUCUGAGCUA 3'	:::: :: ::::.:::::::	query 3' CCGCGAUAGGGAGGACUCGAA 5'
TAS3c	scaffold_37_2031106_2031372_+	12542	88.5%	16078	94.9%	ppt-miR390	4	5-25	target 5' GACGCUACCCUUCCUGAGCUA 3'	: ::::: ::.::::::::	query 3' CCGCGAUAGGGAGGACUCGAA 5'
TAS3d	scaffold_2_4439987_4440271_-	621	96.6%	1268	98.7%	ppt-miR390	0	4-24	target 5' GGCGCUAUCCCUCCUGAGCUU 3'	:::::::::::::::::::::	query 3' CCGCGAUAGGGAGGACUCGAA 5'
TAS3e	scaffold_1_812885_813642_+	305	95.4%	500	84.2%	ppt-miR390	1.5	430-450	target 5' GGCGUUAUCCCUCCUGAGCUG 3'	::::.::::::::::::::::	query 3' CCGCGAUAGGGAGGACUCGAA 5'
TAS6	scaffold_61_112309_112535_+	243	86.0%	720	95.0%	ppt-miR156a	1	890-909	target 5' GUGAUCACUCUCUUCUGUCA 3'	::: ::::::::::::::::	query 3' CACGAGUGAGAGAAGACAGU 5'

Supplemental File S1. Six miRNA binding sites on 6 transcripts with cleavage signals

```
novel-miR3     scaffold_15_2756714_2756873_+_78      Pp1s59_161V6.3 6.5     1124-1143      GTP binding Elongation factor Tu family protein

query   3' CGUGUAUGGUAGUGAGUACUC 5'
           ::.:: ...:::: ::::::
target  5' GCGCAGGTTATCATCATGAACCACCCGGGACAGATCGGTAACGGTTATGCGCCAGTGCTGGATTGCCACACGTCGCACATTGCGGTGAAGTTTGCGGAGATCTTGACGAAGGTGGACAGG 3'
           GCGCAGGTTATCATCATGAACCAC_8
                    ATCATCATGAACCACCCGGGACAG_30
                       ATCATGAACCACCCGGGACAGATC_77
                          ATGAACCACCCGGGACAGATCGGT_20
                                CACCCGGGACAGATCGGTAACGGT_2
                                 ACCCGGGACAGATCGGTAACGGTT_1
                                         CAGATCGGTAACGGTTATGCGCCA_4
                                          AGATCGGTAACGGTTATGCGCCAG_1
                                            ATCGGTAACGGTTATGCGCCAGTG_9
                                                 TAACGGTTATGCGCCAGTGCTGGA_4
                                                                         TTGCCACACGTCGCACATTGCGGT_6
                                                                          TGCCACACGTCGCACATTGCGGTG_1
                                                                              ACACGTCGCACATTGCGGTGAAGT_3
                                                                                        CATTGCGGTGAAGTTTGCGGAGAT_2
                                                                                          TTGCGGTGAAGTTTGCGGAGATCT_9
                                                                                             CGGTGAAGTTTGCGGAGATCTTGA_3
                                                                                              GGTGAAGTTTGCGGAGATCTTGAC_5
                                                                                                      TTGCGGAGATCTTGACGAAGGTGG_7


novel-miR5     scaffold_20_103704_103863_+_20       Pp1s204_5V6.2  5       333-353

query   3' UCUUCACUAGACAUCGCUUCU 5'
           : ::: :::: ::.:::::::
target  5' AAAAGAGATCAGTGGCGAAGACGGACGTGGTTCTGAAAGCTGGGAGTGCAGTGGTGGTGACGGAGGCGCCTCCGCTTTTGAAGACCGCAGAACCCATGCCCATGATGCGCCCTAACAATGG 3'
               GAGATCAGTGGCGAAGACGGACGT_1
                     AGTGGCGAAGACGGACGTGGTTCT_10
                        GGCGAAGACGGACGTGGTTCTGAA_2
                                      TGGTTCTGAAAGCTGGGAGTGCAG_2
                                          TCTGAAAGCTGGGAGTGCAGTGGT_3


novel-miR21    scaffold_66_898001_898160_+_26       Pp1s173_12V6.1 6       2102-2123      SER/ARG-rich protein 34A

query   3' GGUCCAU-GUCACUGAAGCUCA 5'
            .. ::: ::::::.::::.::
target  5' ATGTGTAGCAGTGATTTCGGGTAGAGTTTCAGCATTTGAATGTTCTTGCGTGGTCATATACCCAAATGTGCAACAAGGATTCTAGACACATAATGTTGTGGAAGGTGGTTTTCATGAGAGTA 3'
               GTAGCAGTGATTTCGGGTAGAGTT_2
                      TGATTTCGGGTAGAGTTTCAGCAT_61
                                 AGAGTTTCAGCATTTGAATGTTCT_10
                                    GTTTCAGCATTTGAATGTTCTTGC_1
                                        CAGCATTTGAATGTTCTTGCGTGG_4
                                         AGCATTTGAATGTTCTTGCGTGGT_2
                                           CATTTGAATGTTCTTGCGTGGTCA_3
                                               TGAATGTTCTTGCGTGGTCATATA_2
                                                  ATGTTCTTGCGTGGTCATATACCC_2
                                                      TCTTGCGTGGTCATATACCCAAAT_1
                                                       CTTGCGTGGTCATATACCCAAATG_2
```

```
novel-miR12    scaffold_81_496623_496872_+_599       Pp1s91_206V6.1 7       13-34   DNAJ-like 20

query   3' CGUUCACGUAGAUGU-AGGUGU 5'
           :: :::: ::::.:: :..:::
target  5' GCTAGTGGATCTGCATTTTACATTCTGAAGTGTAGCATTGAGAGAGTGAGCGGAGAGCTGTACGGAGTTGCTCGATTTTTGGTGTAGGAATTGAATTTTAAGATATCGCGGTTGCCCACCTA 3'
                       GCATTTTACATTCTGAAGTGTAGC_16
                                ATTCTGAAGTGTAGCATTGAGAGA_4
                                   CTGAAGTGTAGCATTGAGAGAGTG_1
                                                      GAGTGAGCGGAGAGCTGTACGGAG_2
```

```
novel-miR13    scaffold_99_649008_649257_+_1138      Pp1s29_335V6.1 7.5     1590-1609      prenylated RAB acceptor 1.H

query   3' CCGUGUUGUUAGAUAGUGUCA 5'
            : :::.::.:::. :::::
target  5' TGGACAGCAGTCTGCACAGAGCAAACAAGTACCAGGACTACCAGTGAAGGTAGTAAAGCTGAAACAATAG 3'
                  CAGTCTGCACAGAGCAAACAAGTA_1
                     TCTGCACAGAGCAAACAAGTACCA_23
                          ACAGAGCAAACAAGTACCAGGACT_1
                              AGCAAACAAGTACCAGGACTACCA_2
```

```
novel-miR18    scaffold_83_1142106_1142255_-_725     Pp1s28_211V6.1 8       1582-1602      PRP38 family protein

query   3' UCAAUGGGGUCAGUUAGAUAU 5'
           :: :::  ::::.: :.:.::
target  5' AGCTACAGCAGTTACTTTGTAGTTATTGGCTCGAGAAAATTGGTGAATTGTGATAATTACGCTGGATGGAGGATTATATTTCTTTTTCCTGAGAATTTCCTACGCGGGGAACAATTTATCG 3'
              TACAGCAGTTACTTTGTAGTTATT_3
                    AGTTACTTTGTAGTTATTGGCTCG_1
                      TTACTTTGTAGTTATTGGCTCGAG_153
                                         TCGAGAAAATTGGTGAATTGTGAT_1
                                             GAAAATTGGTGAATTGTGATAATT_1
                                                  TTGGTGAATTGTGATAATTACGCT_1
                                                      TGAATTGTGATAATTACGCTGGAT_1
                                                         ATTGTGATAATTACGCTGGATGGA_3
                                                          TTGTGATAATTACGCTGGATGGAG_1
                                                           TGTGATAATTACGCTGGATGGAGG_1
                                                                 AATTACGCTGGATGGAGGATTATA_1
                                                                  ATTACGCTGGATGGAGGATTATAT_1
                                                                    TACGCTGGATGGAGGATTATATTT_1
                                                                     ACGCTGGATGGAGGATTATATTTC_2
                                                                        CTGGATGGAGGATTATATTTCTTT_1
                                                                                    TTATATTTCTTTTTCCTGAGAATT_1
                                                                                      ATATTTCTTTTTCCTGAGAATTTC_2
                                                                                       TATTTCTTTTTCCTGAGAATTTCC_1
                                                                                         TTTCTTTTTCCTGAGAATTTCCTA_1
                                                                                           TCTTTTTCCTGAGAATTTCCTACG_2
                                                                                             TTTTTCCTGAGAATTTCCTACGCG_2
                                                                                              TTTTCCTGAGAATTTCCTACGCGG_1
                                                                                                 TCCTGAGAATTTCCTACGCGGGGA_1
```

Supplemental File S2. Twenty two targets of siRNAs, their functional annotation and alignment of reads from degradome profiling

```
scaffold_342_334404_334853_+_368_+_389       Pp1s343_50V6.1 4.5     653-673 GroES-like family protein
query   3' AUCUCAGGUGAAGAUUUGGAAG 5'
           :::::  :: ::::.::::::
target  5' TAGAGGCAATTCTGAACCTTAGCCTTAACATTCTAATTAGATGAGCACAGAATTATAACAACCACTTTTCTCGCTTTGTTGACTCTGGATGATACTCTCTTTTTGTATGAATAGTTTGAGT 3'
                      CTGAACCTTAGCCTTAACATTCTA_4
                       TGAACCTTAGCCTTAACATTCTAA_6
                                           CTAATTAGATGAGCACAGAATTAT_1
                                                     GAGCACAGAATTATAACAACCACT_3
                                                                 ATAACAACCACTTTTCTCGCTTTG_4
                                                                      AACCACTTTTCTCGCTTTGTTGAC_4
                                                                              TTCTCGCTTTGTTGACTCTGGATG_1
                                                                                                            TCTTTTTGTATGAATAGTTTGAGT_1
```

```
scaffold_342_334404_334853_+_388_+_126       Pp1s4_450V6.1  5       525-545 DUTP-PYROPHOSPHATASE-LIKE 1
query   3' CGGCUGGAAAGAUAGUUAUUAU 5'
            :::::.:: ::::::: :. :
target  5' TCCGACTTTACTATCAAAGCAGGGGACCGAATCGCCCAACTTATTCTAGAGCTTATCATCACTCCTGATGTGATGGAAGTTGATGATCTAGATGCTACTGCCCGTGGAGACGGTGGATTTG 3'
             CGACTTTACTATCAAAGCAGGGGA_2
                CTTTACTATCAAAGCAGGGGACCG_1
                 TTTACTATCAAAGCAGGGGACCGA_3
                  TTACTATCAAAGCAGGGGACCGAA_2
                       ATCAAAGCAGGGGACCGAATCGCC_19
                                 GGGACCGAATCGCCCAACTTATTC_1
                                           CGCCCAACTTATTCTAGAGCTTAT_1
                                             CCCAACTTATTCTAGAGCTTATCA_2
                                               CAACTTATTCTAGAGCTTATCATC_4
                                                     ATTCTAGAGCTTATCATCACTCCT_7
                                                                 ATCATCACTCCTGATGTGATGGAA_1
                                                                     TCACTCCTGATGTGATGGAAGTTG_2
                                                                             GATGTGATGGAAGTTGATGATCTA_1
                                                                                    TGGAAGTTGATGATCTAGATGCTA_3
                                                                                      GAAGTTGATGATCTAGATGCTACT_1
                                                                                       AAGTTGATGATCTAGATGCTACTG_1
                                                                                          TTGATGATCTAGATGCTACTGCCC_1
                                                                                                ATCTAGATGCTACTGCCCGTGGAG_1
                                                                                                 TCTAGATGCTACTGCCCGTGGAGA_2
                                                                                                     GATGCTACTGCCCGTGGAGACGGT_1
                                                                                                      ATGCTACTGCCCGTGGAGACGGTG_2
                                                                                                       TGCTACTGCCCGTGGAGACGGTGG_1
```

```
scaffold_342_334404_334853_+_388_+_126       Pp1s466_14V6.2 5.5     1539-1559      expansin A1
query   3' CGGCUGGAAAGAUAGUUAUUAU 5'
           :: :.::  ::::::.::.: :
target  5' GCAGGCCGTCTATCGATGAGAAGGTAGCTGCAGTTGTTGGTCTTCTGTAGCGGAAGCCGCTTGCAGGTGACTTCTATTCAGGTGTTGATTGCAATTATTTTTTGATTTATTGGGGGTCTCA 3'
           GCAGGCCGTCTATCGATGAGAAGG_1
                     TATCGATGAGAAGGTAGCTGCAGT_2
                      ATCGATGAGAAGGTAGCTGCAGTT_29
                                   TAGCTGCAGTTGTTGGTCTTCTGT_2
                                                  GTCTTCTGTAGCGGAAGCCGCTTG_1
                                                         GTAGCGGAAGCCGCTTGCAGGTGA_1
                                                                             GTGACTTCTATTCAGGTGTTGATT_1
```

```
scaffold_4_4023891_4025101_+_862_+_136       Pp1s233_42V6.1 5.5     843-863 translation initiation factor 3 subunit H1
query   3' CGUCUAGGAAGGUGCUUACUC 5'
            :: ::::::.::.:.:  ::
target  5' ACAAATCCTTTCATGGAAAAGAATCTGGAATTTTTGATCGAGTGTATGGACGATCTTTCCATGGAGCAGCAGAAGTTCCAATATTATTATCGTAACTTGTCAAGGCAGCAAGTTCAGCAAC 3'
                    TTCATGGAAAAGAATCTGGAATTT_1
                       ATGGAAAAGAATCTGGAATTTTTG_54
                                   TTTGATCGAGTGTATGGACGATCT_2
                                               TATGGACGATCTTTCCATGGAGCA_1
                                                ATGGACGATCTTTCCATGGAGCAG_10
                                                 TGGACGATCTTTCCATGGAGCAGC_1
                                                      GATCTTTCCATGGAGCAGCAGAAG_2
                                                           TTCCATGGAGCAGCAGAAGTTCCA_5
                                                                        CAGAAGTTCCAATATTATTATCGT_9
                                                                                    TATTATTATCGTAACTTGTCAAGG_2
                                                                                     ATTATTATCGTAACTTGTCAAGGC_3
                                                                                      TTATTATCGTAACTTGTCAAGGCA_2
                                                                                       TATTATCGTAACTTGTCAAGGCAG_1
```

```
scaffold_4_4023891_4025101_+_862_+_136       Pp1s76_75V6.1  5       209-228 Ribosomal S17 family protein
query   3' CGUCUAGGAAGGUGCUUACUC 5'
           ::: ::: :::::. .: :::
target  5' GCAATCATTCCATCGAAGAGGCTTCGCAATAAGATCGCAGGATTTGCCACTCATTTGATGAAGCGTATCCAGAGGGGACCAGTGCGTGGAATTTCCCTGAAGTTGCAGGAAGAGGAGCGT 3'
            CAATCATTCCATCGAAGAGGCTTC_1
                  TTCCATCGAAGAGGCTTCGCAATA_3
                   TCCATCGAAGAGGCTTCGCAATAA_1
                      ATCGAAGAGGCTTCGCAATAAGAT_126
                         GAAGAGGCTTCGCAATAAGATCGC_1
                          AAGAGGCTTCGCAATAAGATCGCA_1
                             AGGCTTCGCAATAAGATCGCAGGA_1
                                      AATAAGATCGCAGGATTTGCCACT_3
                                       ATAAGATCGCAGGATTTGCCACTC_2
                                        TAAGATCGCAGGATTTGCCACTCA_3
                                         AAGATCGCAGGATTTGCCACTCAT_5
                                          AGATCGCAGGATTTGCCACTCATT_2
                                           GATCGCAGGATTTGCCACTCATTT_2
                                            ATCGCAGGATTTGCCACTCATTTG_8
                                               GCAGGATTTGCCACTCATTTGATG_3
                                                CAGGATTTGCCACTCATTTGATGA_5
                                                 AGGATTTGCCACTCATTTGATGAA_2
                                                     TTTGCCACTCATTTGATGAAGCGT_2
                                                      TTGCCACTCATTTGATGAAGCGTA_1
                                                       TGCCACTCATTTGATGAAGCGTAT_1
                                                          CACTCATTTGATGAAGCGTATCCA_2
                                                            CTCATTTGATGAAGCGTATCCAGA_1
                                                             TCATTTGATGAAGCGTATCCAGAG_6
                                                              CATTTGATGAAGCGTATCCAGAGG_1
                                                               ATTTGATGAAGCGTATCCAGAGGG_1
                                                                TTTGATGAAGCGTATCCAGAGGGG_3
                                                                 TTGATGAAGCGTATCCAGAGGGGA_4
                                                                  TGATGAAGCGTATCCAGAGGGGAC_3
                                                                   GATGAAGCGTATCCAGAGGGGACC_1
                                                                    ATGAAGCGTATCCAGAGGGGACCA_6
                                                                     TGAAGCGTATCCAGAGGGGACCAG_1
                                                                       AAGCGTATCCAGAGGGGACCAGTG_1
                                                                        AGCGTATCCAGAGGGGACCAGTGC_1
                                                                           GTATCCAGAGGGGACCAGTGCGTG_2
                                                                            TATCCAGAGGGGACCAGTGCGTGG_6
                                                                             ATCCAGAGGGGACCAGTGCGTGGA_14
                                                                              TCCAGAGGGGACCAGTGCGTGGAA_1
                                                                               CCAGAGGGGACCAGTGCGTGGAAT_4
                                                                                CAGAGGGGACCAGTGCGTGGAATT_1
                                                                                 AGAGGGGACCAGTGCGTGGAATTT_10
                                                                                  GAGGGGACCAGTGCGTGGAATTTC_1
                                                                                   AGGGGACCAGTGCGTGGAATTTCC_1
                                                                                    GGGGACCAGTGCGTGGAATTTCCC_1
                                                                                     GGGACCAGTGCGTGGAATTTCCCT_7
                                                                                       GACCAGTGCGTGGAATTTCCCTGA_2
                                                                                        ACCAGTGCGTGGAATTTCCCTGAA_1
                                                                                         CCAGTGCGTGGAATTTCCCTGAAG_1
                                                                                          CAGTGCGTGGAATTTCCCTGAAGT_9
                                                                                           AGTGCGTGGAATTTCCCTGAAGTT_1
                                                                                            GTGCGTGGAATTTCCCTGAAGTTG_1
                                                                                             TGCGTGGAATTTCCCTGAAGTTGC_3
                                                                                                GTGGAATTTCCCTGAAGTTGCAGG_2
                                                                                                   GAATTTCCCTGAAGTTGCAGGAAG_1
```

```
                                                                                                          TCCCTGAAGTTGCAGGAAGAGGAG_4
                                                                                                            CCTGAAGTTGCAGGAAGAGGAGCG_1
                                                                                                             CTGAAGTTGCAGGAAGAGGAGCGT_2
```

```
scaffold_4_4023891_4025101_+_930_+_197       Pp1s72_71V6.1  5       3030-3050      MA3 domain-containing protein
query   3' CGAAUUUUGUCAGAUGAGUUC 5'
           :.:: .:.:::: :.: ::::
target  5' GTTTTGAGCAGTGTGCACAAGTGATTCTCATTTTCACAGACAATCAAGTTTTGTTCGTGAACCGCG 3'
           GTTTTGAGCAGTGTGCACAAGTGA_2
                   CAGTGTGCACAAGTGATTCTCATT_7
                    AGTGTGCACAAGTGATTCTCATTT_2
                     GTGTGCACAAGTGATTCTCATTTT_1
                      TGTGCACAAGTGATTCTCATTTTC_39
                        TGCACAAGTGATTCTCATTTTCAC_1
                           ACAAGTGATTCTCATTTTCACAGA_1
                            CAAGTGATTCTCATTTTCACAGAC_1
                             AAGTGATTCTCATTTTCACAGACA_2
                                TGATTCTCATTTTCACAGACAATC_2
                                   TTCTCATTTTCACAGACAATCAAG_2
                                    TCTCATTTTCACAGACAATCAAGT_1
                                     CTCATTTTCACAGACAATCAAGTT_1
                                      TCATTTTCACAGACAATCAAGTTT_2
                                        ATTTTCACAGACAATCAAGTTTTG_4
                                         TTTTCACAGACAATCAAGTTTTGT_3
                                          TTTCACAGACAATCAAGTTTTGTT_4
                                           TTCACAGACAATCAAGTTTTGTTC_1
                                              ACAGACAATCAAGTTTTGTTCGTG_4
                                               CAGACAATCAAGTTTTGTTCGTGA_1
                                                AGACAATCAAGTTTTGTTCGTGAA_3
```

```
scaffold_4_4023891_4025101_+_931_+_158       Pp1s101_2V6.1  5      528-547 photosystem I subunit E-2
query   3' CCGAAUUUUGUCAGAUGAGUU 5'
            :::: .:.:::::::.: .::
target  5' AGCTTGAGCAGTCTGCCTAAACTCTCTGTAACTTTCTCATGTCTTTGCCTTAAGATATACTCGCTGAGAATGTATTTATAAATGTTACTATCTTATTTTTCGCGATAGATACTGTGAATC 3'
           AGCTTGAGCAGTCTGCCTAAACTC_6
            GCTTGAGCAGTCTGCCTAAACTCT_9
                 AGCAGTCTGCCTAAACTCTCTGTA_9
                   CAGTCTGCCTAAACTCTCTGTAAC_110
                      TCTGCCTAAACTCTCTGTAACTTT_533
                        TGCCTAAACTCTCTGTAACTTTCT_40
                          CCTAAACTCTCTGTAACTTTCTCA_4
                              AACTCTCTGTAACTTTCTCATGTC_9
                                CTCTCTGTAACTTTCTCATGTCTT_53
                                          CTTTCTCATGTCTTTGCCTTAAGA_18
                                           TTTCTCATGTCTTTGCCTTAAGAT_39
                                            TTCTCATGTCTTTGCCTTAAGATA_43
                                               TCATGTCTTTGCCTTAAGATATAC_72
                                                CATGTCTTTGCCTTAAGATATACT_42
                                                      TTTGCCTTAAGATATACTCGCTGA_44
                                                       TTGCCTTAAGATATACTCGCTGAG_45
                                                              AAGATATACTCGCTGAGAATGTAT_24
                                                                                 TGTATTTATAAATGTTACTATCTT_1
                                                                                  GTATTTATAAATGTTACTATCTTA_7
                                                                                     TTTATAAATGTTACTATCTTATTT_20
                                                                                              GTTACTATCTTATTTTTCGCGATA_10
                                                                                                       TTATTTTTCGCGATAGATACTGTG_1
                                                                                                         ATTTTTCGCGATAGATACTGTGAA_11

TAS3a_421_-_129       Pp1s222_28V6.1 5.5     77-99
query   3' CCGCUUUUAUCCA-CUCGACCCC 5'
           ::.:.:.:: ::: ::: ::::
target  5' GGTGGAGATTGGTAGAGATGGGTGGAGGCATCGCCAGGCCCACGCCCTTGCAGCAAGTGGGTGCCGCGGCTGCACCGAGCCGTGATTCCAGCGCGACATTCAATATTCAGTCCATCGTCGTGA 3'
           GGTGGAGATTGGTAGAGATGGGTG_1
                        AGAGATGGGTGGAGGCATCGCCAG_157
                         GAGATGGGTGGAGGCATCGCCAGG_17
                          AGATGGGTGGAGGCATCGCCAGGC_1
                            ATGGGTGGAGGCATCGCCAGGCCC_9
                              GGGTGGAGGCATCGCCAGGCCCAC_10
                                    AGGCATCGCCAGGCCCACGCCCTT_1
                                                                                            GTGATTCCAGCGCGACATTCAATA_3
                                                                                                    AGCGCGACATTCAATATTCAGTCC_1

TAS3a_448_+_984       Pp1s143_131V6.4       5.5    2001-2022      membrane protein, putative
query   3' ACGAA-UUGUGGGGCGAUUGUU 5'
           : ::: .::::: ::::: ..:
target  5' TTCTTCGACACCACGCTACTGAACTTGGGGTAGTGGTGCCACTAGCGGCCATCTTCTCCATCCTGACCACTGTAATTGCGGGACGTCAACTAGGTCTTCAAGAATTTCTCACACGCTCAGTC 3'
                       ACGCTACTGAACTTGGGGTAGTGG_9
                        CGCTACTGAACTTGGGGTAGTGGT_2
                                    TGGGGTAGTGGTGCCACTAGCGGC_1
                                                               CTTCTCCATCCTGACCACTGTAAT_6
                                                                      ATCCTGACCACTGTAATTGCGGGA_41
                                                                                   TAATTGCGGGACGTCAACTAGGTC_8
                                                                                    AATTGCGGGACGTCAACTAGGTCT_1
                                                                                        GCGGGACGTCAACTAGGTCTTCAA_1
                                                                                                      AGGTCTTCAAGAATTTCTCACACG_1
                                                                                                         TCTTCAAGAATTTCTCACACGCTC_10
```

```
TAS3a_449_+_236        Pp1s360_33V6.1 5       1396-1416      Chaperone DnaJ-domain superfamily protein
query   3' UUACGAAUUGUGGGGCGAUUGU 5'
           :::: : :.:: :.::: ::::
target  5' AATGATCAGCAGCTCGCAACAGGTTACTCGGAAATGTTCAATTCTGCTGGTAACATATGTAGTCCATTTCGGATGGATTTCGTGTTGGTTT 3'
           AATGATCAGCAGCTCGCAACAGGT_1
             TGATCAGCAGCTCGCAACAGGTTA_1
               ATCAGCAGCTCGCAACAGGTTACT_2
                TCAGCAGCTCGCAACAGGTTACTC_1
                    CAGCTCGCAACAGGTTACTCGGAA_4
                       CTCGCAACAGGTTACTCGGAAATG_1132
                          GCAACAGGTTACTCGGAAATGTTC_1
                              CAGGTTACTCGGAAATGTTCAATT_1
                                 GTTACTCGGAAATGTTCAATTCTG_1
                                  TTACTCGGAAATGTTCAATTCTGC_2
                                   TACTCGGAAATGTTCAATTCTGCT_8
                                     CTCGGAAATGTTCAATTCTGCTGG_2
                                      TCGGAAATGTTCAATTCTGCTGGT_1
                                                     TCTGCTGGTAACATATGTAGTCCA_1
                                                       TGCTGGTAACATATGTAGTCCATT_1
                                                         CTGGTAACATATGTAGTCCATTTC_2
                                                          TGGTAACATATGTAGTCCATTTCG_1
                                                           GGTAACATATGTAGTCCATTTCGG_1
                                                            GTAACATATGTAGTCCATTTCGGA_5
                                                              AACATATGTAGTCCATTTCGGATG_1
                                                                 ATATGTAGTCCATTTCGGATGGAT_1
                                                                  TATGTAGTCCATTTCGGATGGATT_3
                                                                   ATGTAGTCCATTTCGGATGGATTT_2
                                                                    TGTAGTCCATTTCGGATGGATTTC_4
                                                                      TAGTCCATTTCGGATGGATTTCGT_2
                                                                        GTCCATTTCGGATGGATTTCGTGT_1
                                                                         TCCATTTCGGATGGATTTCGTGTT_2

TAS3a_467_-_131        Pp1s52_243V6.2 5.5     1695-1716      Cytokine-induced anti-apoptosis inhibitor 1, Fe-S biogenesis
query   3' GUAAACUUACGUUGUGA-GGAU 5'
           :: ::::: :::.. :: :::.
target  5' CAATTGAAAGCAGTTCTACCTGTAGTTACATGATGCAATAACATGCTAAGCTCCTTTCATAAGTTTCTATCATGTTCCTCATAGATATCTGCTGCAGAAGCTACTATTAATCGATCATCTTG 3'
              TTGAAAGCAGTTCTACCTGTAGTT_2
                 AAAGCAGTTCTACCTGTAGTTACA_1
                        TTCTACCTGTAGTTACATGATGCA_716
                                   GTTACATGATGCAATAACATGCTA_2
                                       CATGATGCAATAACATGCTAAGCT_1
                                        ATGATGCAATAACATGCTAAGCTC_1
                                          GATGCAATAACATGCTAAGCTCCT_10
                                            TGCAATAACATGCTAAGCTCCTTT_4
                                               AATAACATGCTAAGCTCCTTTCAT_5
                                                  AACATGCTAAGCTCCTTTCATAAG_1
                                                    CATGCTAAGCTCCTTTCATAAGTT_2
                                                                   TCATAAGTTTCTATCATGTTCCTC_1
                                                                    CATAAGTTTCTATCATGTTCCTCA_1
                                                                          TTTCTATCATGTTCCTCATAGATA_9
                                                                                TCATGTTCCTCATAGATATCTGCT_6
                                                                                                  TCTGCTGCAGAAGCTACTATTAAT_13
                                                                                                    TGCTGCAGAAGCTACTATTAATCG_3
                                                                                                        GCAGAAGCTACTATTAATCGATCA_2
                                                                                                         CAGAAGCTACTATTAATCGATCAT_10
                                                                                                          AGAAGCTACTATTAATCGATCATC_6
```

```
TAS3a_472_-_209       Pp1s321_30V6.1 5.5     3090-3110      H(+)-ATPase 6
query   3' CUUACGUUGUGAGGAUGCGUU 5'
            .:: ::.:: :::::: ::.
target  5' TGATCCAGCAGTCCTACACAGTCTAAAGCACCAGAAGCAAATGTGCTAAGAGTCTAATTCAGTCTTGGCCTAGTTTTAGTAATTCTGTTTACTGTCGCCATAAAAAGCTTAGCAATGAGAA 3'
              TCCAGCAGTCCTACACAGTCTAAA_1
                      TCCTACACAGTCTAAAGCACCAGA_14
                             CAGTCTAAAGCACCAGAAGCAAAT_1
                                TCTAAAGCACCAGAAGCAAATGTG_1
                                  TAAAGCACCAGAAGCAAATGTGCT_1
                                     AGCACCAGAAGCAAATGTGCTAAG_1
                                           AGAAGCAAATGTGCTAAGAGTCTA_1
                                                            GAGTCTAATTCAGTCTTGGCCTAG_1
                                                                        GTCTTGGCCTAGTTTTAGTAATTC_1
                                                                                 TAGTTTTAGTAATTCTGTTTACTG_2
                                                                                         GTAATTCTGTTTACTGTCGCCATA_1
                                                                                          TAATTCTGTTTACTGTCGCCATAA_1


TAS3a_472_-_209       Pp1s221_76V6.1 5.5     1188-1209      photosystem II BY
query   3' CUUAC-GUUGUGAGGAUGCGUU 5'
            .::: .:: ::::::::: :.
target  5' CGATGTTAAAACTCCTACGAAGGGTGGAAAGAGATTCAAGAGCGTCATCGGAGCCACCGGAATCAGCTTAGCTGCAAGCTCACTGTTGGCTGCCCCTGAAGCCGATGCTCAGGTGCAAGAGA 3'
                      CTCCTACGAAGGGTGGAAAGAGAT_12
                                 GGTGGAAAGAGATTCAAGAGCGTC_4
                                           GATTCAAGAGCGTCATCGGAGCCA_14
                                                                         TCAGCTTAGCTGCAAGCTCACTGT_10
                                                                            GCTTAGCTGCAAGCTCACTGTTGG_12
                                                                                    GCAAGCTCACTGTTGGCTGCCCCT_1
                                                                                              TGTTGGCTGCCCCTGAAGCCGATG_2


TAS3a_485_-_281       Pp1s234_30V6.7 5.5     2792-2810      Major facilitator superfamily protein
query   3' GAUGCGUUCUGGGAUCGAUA 5'
           .:: ::::  .:::::.::
target  5' TTAGCAATCTCCTAGTTAGAATTGTTGTGTCGGAAATCTGACAATTATTTCGTAATCAGATGCTACATTATAGCATTACCTTCGAGTAGAGTCGACTTGACATGTTTACATAGTTGCA 3'
           TTAGCAATCTCCTAGTTAGAATTG_1
                      CTAGTTAGAATTGTTGTGTCGGAA_18
                       TAGTTAGAATTGTTGTGTCGGAAA_2
                                                                           TACATTATAGCATTACCTTCGAGT_1
                                                                                TATAGCATTACCTTCGAGTAGAGT_2
                                                                                                          ACTTGACATGTTTACATAGTTGCA_1


TAS3a_508_+_477       Pp1s68_202V6.3 5.5     917-938 outer plastid envelope protein 16-1
query   3' CGUG-AGUAGUGUGGGAUACCU 5'
            :.: ::::.::::  :.::::
target  5' ACGCTTCATTACACGGTGTGGAAATCGTCTTCGGCTTGAGCCATTACTTTATTACAATGTGGCTAAATTGCCTGTAACAAGTATGTGTAAACCTCTTACGTGGCTGTTGAAAGTTTTATTTC 3'
                       ACGGTGTGGAAATCGTCTTCGGCT_54
                          GTGTGGAAATCGTCTTCGGCTTGA_9
                                              TTGAGCCATTACTTTATTACAATG_10
                                                                         CTAAATTGCCTGTAACAAGTATGT_3
                                                                                       ACAAGTATGTGTAAACCTCTTACG_8
                                                                                          AGTATGTGTAAACCTCTTACGTGG_1
                                                                                           GTATGTGTAAACCTCTTACGTGGC_10
                                                                                                    AACCTCTTACGTGGCTGTTGAAAG_4
```

```
TAS3a_536_+_115       Pp1s255_54V6.1 4.5     1613-1632      GroES-like zinc-binding alcohol dehydrogenase family protein
query   3' GUUCCGUCAUCUUCUCGUGG 5'
            : ::::: ::::: ::::.:
target  5' GATGGCAGCAGAAGCGCATCGAGCAAGGGATAGCAAGAATGCTGGTGGTAAAGTTGTTCTAAAAGTTGATGTTTAGGTGAACGTGCAGTATTGAAGTACTATACAGACATCATATCTATA 3'
                      AAGCGCATCGAGCAAGGGATAGCA_260
                                AGCAAGGGATAGCAAGAATGCTGG_2
                                                                               ATGTTTAGGTGAACGTGCAGTATT_1
                                                                                       GTGAACGTGCAGTATTGAAGTACT_4
                                                                                          AACGTGCAGTATTGAAGTACTATA_2
                                                                                                   TATTGAAGTACTATACAGACATCA_13
                                                                                                           TACTATACAGACATCATATCTATA_1

TAS3a_536_+_115       Pp1s143_106V6.1        4.5     600-620 ATPase, F0/V0 complex, subunit C protein
query   3' GU-UCCGUCAUCUUCUCGUGG 5'
           :: :::::::::  : ::::.
target  5' CATAGGCAGTAGCTGTGCACTTTCAGATGCTCAGAACTCCACATTGTTTGTCAAGATTCTAGTTATTGAGATTTTTGGCAGCGCTCTAGGTTTGTTTGGAGTTATTGTAGGTATCATCATG 3'
              AGGCAGTAGCTGTGCACTTTCAGA_1
                      GCTGTGCACTTTCAGATGCTCAGA_1
                       CTGTGCACTTTCAGATGCTCAGAA_172
                        TGTGCACTTTCAGATGCTCAGAAC_1
                               TTTCAGATGCTCAGAACTCCACAT_1
                                                     ATTGTTTGTCAAGATTCTAGTTAT_1
                                                                          TATTGAGATTTTTGGCAGCGCTCT_1
                                                                                             GCTCTAGGTTTGTTTGGAGTTATT_1
                                                                                                  AGGTTTGTTTGGAGTTATTGTAGG_3
                                                                                                    GTTTGTTTGGAGTTATTGTAGGTA_3

TAS3a_601_+_111       Pp1s161_32V6.2 4.5     78-96   photosystem I light harvesting complex gene 1
query   3' UCACUAUAUGUUGGCUAUGU 5'
           . : .:.:::::::::: ::
target  5' GATCGTGTACAACCGATCAATCTAGTCGCAGAAATGGCGTCCGCAATGGCCACTGCTGCCGCCCATGTGAGCTGTGTGGTGACTGGAGTCGCCACTGCCCAAAAGTCCAGCTTCTTCGC 3'
                   ACAACCGATCAATCTAGTCGCAGA_29
                            CAATCTAGTCGCAGAAATGGCGTC_1
                                  AGTCGCAGAAATGGCGTCCGCAAT_3
                                              GGCGTCCGCAATGGCCACTGCTGC_2
                                                                          CATGTGAGCTGTGTGGTGACTGGA_3
                                                                                            ACTGGAGTCGCCACTGCCCAAAAG_2
                                                                                             CTGGAGTCGCCACTGCCCAAAAGT_1

TAS3c_100_-_273       Pp1s109_232V6.2        4.5     255-275
query   3' GAAUUCAGAUUCCUUAUCUAAU 5'
           :::. : : ::::::::: ::
target  5' CTTGTGCCAAGGAATAGACTATCACCAAACATGATCGATAGATCAGGAGTGTAGACAAAAAAATATATATCTAAAAAAGAAAAGAAAAAAAGGTCCATGTACCACTTACATGAATCTGTAT 3'
                GCCAAGGAATAGACTATCACCAAA_72
                 CCAAGGAATAGACTATCACCAAAC_126
                      GAATAGACTATCACCAAACATGAT_5969
                       AATAGACTATCACCAAACATGATC_480
                          AGACTATCACCAAACATGATCGAT_2158
                                       ACATGATCGATAGATCAGGAGTGT_3
                                              CGATAGATCAGGAGTGTAGACAAA_4
                                               GATAGATCAGGAGTGTAGACAAAA_17
                                                                               ATCTAAAAAAGAAAAGAAAAAAAG_1
                                                                                                   AAAGGTCCATGTACCACTTACATG_1
                                                                                                        TCCATGTACCACTTACATGAATCT_7
                                                                                                          CATGTACCACTTACATGAATCTGT_90
```

```
TAS3a_536_+_115        Pp1s3_368V6.1  5.5    429-448 Histone superfamily protein
query   3' GUUCCGUCAUCUUCUCGUGG 5'
            .:::::: :::.:  .:::
target  5' GGAGGCAGCAGAGGCCTACCTGGTTGGTTTGTTTGAGGATACCAACTTGTGTGCCATCCATGCAAAGCGGGTGACAATTATGCCCAAAGACATCCAACTGGCCAGGAGAATCAGGGGAGA 3'
            GAGGCAGCAGAGGCCTACCTGGTT_3
             AGGCAGCAGAGGCCTACCTGGTTG_5
              GGCAGCAGAGGCCTACCTGGTTGG_3
                  GCAGAGGCCTACCTGGTTGGTTTG_1
                      AGGCCTACCTGGTTGGTTTGTTTG_463
                           TACCTGGTTGGTTTGTTTGAGGAT_2
                                   TGGTTTGTTTGAGGATACCAACTT_5
                                     GTTTGTTTGAGGATACCAACTTGT_5
                                      TTTGTTTGAGGATACCAACTTGTG_20
                                       TTGTTTGAGGATACCAACTTGTGT_8
                                        TGTTTGAGGATACCAACTTGTGTG_9
                                          TTTGAGGATACCAACTTGTGTGCC_16
                                            TGAGGATACCAACTTGTGTGCCAT_5
                                             GAGGATACCAACTTGTGTGCCATC_2
                                               GGATACCAACTTGTGTGCCATCCA_2
                                                     CAACTTGTGTGCCATCCATGCAAA_2
                                                      AACTTGTGTGCCATCCATGCAAAG_8
                                                       ACTTGTGTGCCATCCATGCAAAGC_1
                                                          TGTGTGCCATCCATGCAAAGCGGG_17
                                                           GTGTGCCATCCATGCAAAGCGGGT_30
                                                            TGTGCCATCCATGCAAAGCGGGTG_21
                                                                 CATCCATGCAAAGCGGGTGACAAT_6
                                                                    CCATGCAAAGCGGGTGACAATTAT_1
                                                                      ATGCAAAGCGGGTGACAATTATGC_35
                                                                       TGCAAAGCGGGTGACAATTATGCC_3
                                                                          AAAGCGGGTGACAATTATGCCCAA_5
                                                                            AGCGGGTGACAATTATGCCCAAAG_4
                                                                              CGGGTGACAATTATGCCCAAAGAC_1
                                                                                GGTGACAATTATGCCCAAAGACAT_1
                                                                                 GTGACAATTATGCCCAAAGACATC_20
                                                                                  TGACAATTATGCCCAAAGACATCC_10
                                                                                   GACAATTATGCCCAAAGACATCCA_8
                                                                                    ACAATTATGCCCAAAGACATCCAA_5
                                                                                      AATTATGCCCAAAGACATCCAACT_9
                                                                                          ATGCCCAAAGACATCCAACTGGCC_5
                                                                                                      ATCCAACTGGCCAGGAGAATCAGG_9
                                                                                                        CCAACTGGCCAGGAGAATCAGGGG_3
                                                                                                         CAACTGGCCAGGAGAATCAGGGGA_3
```

```
TAS3c_111_+_225       Pp1s86_156V6.2 5      1431-1450      glyceraldehyde-3-phosphate dehydrogenase B subunit
query   3' GGUGGGAUGGAUUAGAUAAGG 5'
           .:..:: :.::::::::: :
target  5' TCGTCCATCTAATCTATCCGCCACCTCGAAAATGGGAATTTTTTTTCTTACTTCAACTGCC 3'
                    TAATCTATCCGCCACCTCGAAAAT_78
                           TCTATCCGCCACCTCGAAAATGGG_56
                                 CGCCACCTCGAAAATGGGAATTTT_1



TAS3d_3_-_1127 Pp1s47_90V6.1  5      1123-1143      Homeodomain-like superfamily protein
query   3' CCGCGAUAGGGAGGACUCGAA 5'
           ::. . : ::::::::::::
target  5' GGTTTGAACCCTCCTGAGCTATCACACAAACATGGAACAGCCGACTCGCAAGGCGATATCCCACATGCTGCCCACAATCTGCAGCAGCAGCAGTCGCGCGTACAGTCAGGCGCCGTGTCCC 3'
               TGAACCCTCCTGAGCTATCACACA_1
                      TCCTGAGCTATCACACAAACATGG_33
                                  ACACAAACATGGAACAGCCGACTC_1
                                                     CTCGCAAGGCGATATCCCACATGC_1
```
